# Counting missing babies in Tanzania: Neonatal mortality data quality from Tanzania’s District Health Information System across 28 Regional and 7 Tertiary hospitals (2015–2024)

**DOI:** 10.1371/journal.pone.0348874

**Published:** 2026-07-23

**Authors:** Josephine Shabani, Nahya Salim, Lucas Malla, James H. Cross, Rebecca E. Penzias, Audêncio Victor, Christine Bohne, Ahmad Mohamed Makuwani, Habib Ismail, Felix Bundala, Claud Kumalija, Morris Ondieki, Prosper Mshana, Jacqueline Minja, Irabi Kassim, Jennie Jaribu, Honorati Masanja, Eric O. Ohuma, Joy E. Lawn

**Affiliations:** 1 Ifakara Health Institute, Dar es Salaam, Tanzania; 2 Faculty of Epidemiology and Population Health, London School of Hygiene & Tropical Medicine, London, United Kingdom; 3 Department of Pediatrics and Child Health, Muhimbili University of Health and Allied Sciences (MUHAS), Dar es Salaam, Tanzania; 4 School of Public Health, University of São Paulo (USP), São Paulo, São Paulo, Brazil; 5 Rice360 Institute for Global Health Technologies, Rice University, Houston, Texas, United States of America; 6 Ministry of Health, Dodoma, Tanzania; LMU München: Ludwig-Maximilians-Universitat Munchen, NEPAL

## Abstract

**Introduction:**

The Sustainable Development Goal 3.2 targets reducing the neonatal mortality rate (NMR) to <12 per 1,000 live births by 2030; however, 65 countries, including Tanzania, remain off track. The 2022/23 Tanzania Demographic and Health Survey (TDHS) reported an NMR of 24 per 1,000 live births, equivalent to 48,000 neonatal deaths annually.

**Methods:**

Neonatal deaths were extracted from the District Health Information System (DHIS2) for 28 Regional Referral Hospitals (RRHs) and seven tertiary hospitals from 2015 to 2024. Data quality was assessed by: (1) completeness, as the proportion of months each hospital reported neonatal deaths; (2) internal consistency, comparing mean NMR for 2021–2023 with 2024 following WHO guidance; and (3) external plausibility, comparing DHIS2 with DHS and UN estimates. For seven hospitals implementing NEST360 program, NMRs from DHIS2 were compared with the Neonatal Inpatient Dataset (NID) for 2021 and 2024.

**Results:**

Between 2015 and 2024, DHIS2 recorded an average of >8,500 neonatal deaths annually versus 48,000 expected. RRHs accounted for 30–40% of reported deaths, and tertiary hospitals for 4–11%. Overall monthly reporting completeness was 58.3%, higher in RRHs (median 61.7%, IQR 8.3–95.0%) than in tertiary hospitals (median 50%, IQR 0.8–73.3%), with only 8 RRHs (28.5%) and no tertiary hospital achieving >75% reporting. Only 28.6% of hospitals had internal consistency (NMR within ±33% of the mean) for 2021–2023. The DHIS2 NMR for 2024 was 5.9 per 1,000 (adjusted for ~20% home births), compared to 24 per 1,000 in DHS 2022/23. Compared with NEST360 NID, one tertiary hospital reported lower DHIS2 NMRs, and three RRHs reported higher (ratio >1).

**Conclusion:**

Neonatal mortality reporting in Tanzania’s DHIS2 remains substantially under-captured, with notable variations across facilities. Although RRHs perform better than tertiary hospitals, overall data quality remains low. Improving reporting in underperforming facilities and leveraging high-performing RRHs can strengthen national neonatal mortality monitoring.

## Introduction

Newborn deaths remain a major public health concern, with an estimated 2.3 million newborn deaths worldwide in 2023 [1–5]. While the global community has made remarkable steps in reducing under-five mortality between 1990 and 2023, the pace of decline in newborn deaths has consistently lagged behind that of post-neonatal child mortality, making newborns an increasingly dominant share of all child deaths [2,6]. The 2022/23 Tanzania Demographic and Health Survey (TDHS) reported a Neonatal Mortality Rate (NMR) of 24 per 1,000 live births, translating to approximately 48,000 neonatal deaths annually [7]. Tanzania has committed to achieving Sustainable Development Goal (SDG) 3.2, to reduce the NMR to less than 12 per 1,000 live births by 2030 [2]. This SDG commitment is reflected in the Tanzania Development Vision 2050, the Health Sector Strategic Plan (HSSP V 2021–2025), and the National Plan for Reproductive, Maternal, Newborn, Child and Adolescent Health and Nutrition, 2021–2026 (One Plan III) [8–11].

Although the percentages of births in health facilities have increased from 64% in the 2015/16 TDHS to 81% in the 2022/23 TDHS [7,12], there has been no significant change in NMR between the 2010 TDHS, 2015/16 TDHS, and 2022/23 TDHS. Eighty per cent (80%) of newborn deaths in Tanzania are attributed to three main conditions: preterm births, intrapartum complications, and infections [13–17]. These deaths can be prevented by high coverage and quality of neonatal care interventions, implementing World Health Organisation (WHO) Level 2 care including Kangaroo Mother Care (KMC) for preterm babies [18,19], feeding, respiratory support with Continuous Positive Airway Pressure (CPAP), infection prevention and control, early detection and treatment of sepsis, and proper management of neonatal jaundice [4,7,9,20]. Improving maternal and newborn care is essential for more effectively reducing these deaths.

DHIS2 (District Health Information Software 2) is an open-source, web-based platform specifically designed for managing and reporting health data [21,22]. It is widely used in low- and middle-income countries to collect, manage, analyse, and visualise health-related information. Its implementation aims to strengthen accessibility, availability, and utilisation of reliable health data for decision-making at both national and subnational levels [23,24]. In Tanzania, DHIS2 serves as Tanzania’s primary platform for collating and reporting health data from hospital registers across different levels of healthcare facilities [25].

A systematic review on the quality of health facility data regarding newborn indicators in Low and Middle-Income Countries (LMICs) revealed that while high-quality data collection is achievable in certain high-burden LMIC settings, there remains a significant gap in the published literature and robust evidence needed to drive policy initiatives aimed at improving newborn health metrics [22,26]. Addressing this gap is essential for meeting global health targets. Common data quality issues, such as incompleteness and inconsistency, have been identified across various DHIS2 studies in Tanzania [27–31]. However, several data quality initiatives, including supportive supervision, data quality assessments (DQA), and data use workshops, have been implemented at various levels [29,32].

Over the past two decades, Tanzania has achieved considerable reductions in child mortality, alongside recent notable declines in maternal mortality [7], prompting a strategic shift toward national and subnational interventions focused on reducing neonatal mortality [16]. During the late Millennium Development Goals (MDG) era, the increase in facility births led to the national scale-up of neonatal resuscitation programs, such as Helping Babies Breathe. Additionally, the WHO’s Integrated Management of Childhood Illness (IMCI) framework, which standardises the management and prevention of childhood illnesses, was expanded to include a newborn component. Since 2010, KMC has also been scaled up nationally and incorporated into many district health budgets [33]. Despite these advancements, there remains a recognised gap for a more comprehensive small and sick newborn care package. The government has developed norms for this package and is now committed to scaling it nationally [34]. However, the success of these initiatives at scale depends on the availability of high-quality DHIS2 data for effective monitoring and evaluation [24,35,36].

This paper builds on a previous study’s broader findings, which evaluated neonatal indicators in DHIS2 across over 7,000 health facilities in Tanzania from 2015 to 2022. The study found that although 70% of Every Newborn Action Plan (ENAP) indicators were recorded, their reliability was a major concern. Specifically, maternal and newborn outcomes, including complications such as stillbirths, were underreported. The study found that the neonatal mortality rate (NMR) was underestimated by more than 75% [30]. This finding highlights a crucial gap in the accuracy of neonatal data. Building on this, the current study focuses on higher-level facilities where the majority of neonatal deaths are reported to occur [15,37]. This study contributes novel insights into these data quality challenges, particularly within the context of regional and tertiary neonatal care.

For this analysis, the national neonatal mortality rate refers to deaths in the first 28 days after birth per 1000 live births, as captured by national health surveys such as DHS. Inpatient neonatal mortality rate is the official DHIS2 and WHO indicator. It refers to the deaths of neonates during admission (so this may not include to 28 days) amongst those admitted to health care facilities [38]. This study aims to evaluate the availability and quality of hospital neonatal mortality data in Tanzania from 2015 to 2024 using DHIS2 in RRHs and tertiary hospitals. Additionally, the study utilised neonatal inpatient data (NID) with Newborn Essential Solutions and Technologies (NEST360), an alliance involving five African countries (Ethiopia, Kenya, Malawi, Nigeria, and Tanzania), to compare inpatient NMRs across the seven NEST360 implementing hospitals in Tanzania between 2021 and 2024. NEST360 aims to reduce neonatal inpatient deaths by improving level-2 newborn care through device installation, training, and quality improvement initiatives [39]. NEST360 specifically captures newborns admitted to the Neonatal Care Units (NCUs) and may miss the smaller number who die quickly on the labour ward.

The specific objectives are:

1. Evaluating the quality of neonatal death data reported by 28 regional referral hospitals (RRHs) and 7 tertiary hospitals from 2015 to 2024.
2. Comparing inpatient neonatal mortality rates from DHIS2 with NEST360 Neonatal Inpatient Database (NID) across seven hospitals between 2021–2024.

## Materials and methods

### Study settings

The analysis focused on Tanzania’s mainland, which had an estimated population of 68.6 million in 2024 [40], distributed across eight zones, 26 regions, and 184 districts [7]. Health services in Tanzania are delivered through both public and private healthcare facilities. Each region has one regional referral hospital, except for Dar-es-Salaam, which has three. The health system in Tanzania is primarily organised into a district-level system, with a strong emphasis on primary healthcare. The structure of the health system is based on a three-tier system:

Level 1 (primary) includes i) community services provided by community health workers (CHWs) and are responsible for delivering essential health education and promotion and some curative services directly within the community, ii) dispensary – is the lowest level typically serving one or a few villages or a ward and primarily focuses on providing out-patient care, iii) health centre – offer a higher level of care compared to the dispensary and serves a larger population and is also required to provide inpatient care, often receiving referrals from the nearest dispensaries, and iv) district hospital or a designated district hospital located at a district council – serves as the primary referral within the district and provides more comprehensive healthcare services.

Level 2 (secondary) includes regional referral hospitals, which provide specialist medical care and serve as referral centres for patients requiring advanced medical services beyond the capabilities of primary healthcare facilities.

Level 3 (tertiary) includes zonal and national hospitals, which offer advanced medical care and serve as teaching hospitals for medical, paramedical, and nursing training. These facilities provide specialised services and are equipped to handle advanced medical care [4] (S1 Fig).

This study will focus on secondary and tertiary levels, including 28 regional referral hospitals and seven tertiary-level hospitals. The tertiary-level hospitals include five zonal hospitals, one national hospital and one super-specialist national hospital. Additionally, the study will also focus on seven NEST360 implementation hospitals in Tanzania, which are also part of RRHs and tertiary hospitals. Ethical approval for the NEST360 program was obtained from the local institutional review board, National Institute for Medical Research (NIMR) Tanzania (NIMR/HQ/R.8c/Vol.I/3037, The study primarily involved secondary analysis of DHIS2 data (https://dhis2.org/). Since DHIS2 data consist of routinely collected service statistics, individual consent was not required. No identifiable information, such as personal names, was included in this work.

### Data sources

By 2024, there were 11,211 health facilities registered in Tanzania’s health facility registry. This includes 466 hospitals (1 national hospital, 1 super-specialist national hospital, 5 zonal referral hospitals, 28 regional referral hospitals, 184 district hospitals, and 246 other hospitals, including private for-profit and faith-based hospitals), 1,278 health centres, and 8,437 dispensaries.

The national rollout of the DHIS2 started in 2012, and by December 2013, all districts in the country were using the system. Since the rollout of DHIS2 in Tanzania, the number of facilities actively reporting to the system has increased; currently, 98% of facilities report data, with hospitals reporting the highest (92.9%) [41,42].

We analysed neonatal death data from DHIS2 using the hospital as the unit of analysis. The aggregate DHIS2 data is available on a government portal for registered users with login credentials. The first author (JS) has access through an existing agreement between the Government and Ifakara Health Institute, where JS is based. Similarly, the Neonatal Inpatient Data (NID) is available through Ifakara Health Institute, one of the collaborating sites for NEST360 and responsible for data collection and management. NID was codesigned as a parsimonious individual-level dataset for quality of neonatal care and includes variables covering clinical care, interventions, selected investigations, and discharge outcomes [43].

We assigned codes RRH1-RRH28 to represent 28 regional referral hospitals, and tertiary1- tertiary7 to represent the 7 tertiary hospitals. The data were compared and presented through tables and figures for visualisation. Analysis was performed using Stata version 18 (StataCorp LLC, Texas, USA).

### Data quality dimensions and analysis

The data was analysed according to three quality dimensions adapted from the WHO data quality assessment framework: (1) completeness of neonatal mortality data element, (2) internal consistency, and (3) plausibility comparison whereby we compared neonatal mortality rates from DHIS2 to the Tanzania DHS estimates [7] and United Nations Inter-agency Group for Child Mortality Estimation (UN-IGME) estimates [2].

### Quality of neonatal deaths reporting by 28 Regional Referral Hospitals (RRHs) and seven tertiary hospitals over the period 2015–2024 using DHIS2 data

#### Completeness of neonatal death reporting.

To assess completeness, we calculated the percentage of months in a year in which hospitals reported mortality data. Over 10 years, each facility was expected to report data for 120 months, so monthly completeness was determined by dividing the total number of months a facility reported by 120. Overall, across the 35 facilities, a total of 4200 facility-months was expected (35 facilities x 120 months). The following cut-off points (<25%, 25–50%, 50–75%, and>=75%) were used to measure the extent of complete reporting. Facilities with a completeness rate of 75% or higher are considered to have high reporting. We also calculated the percentage of facilities reporting neonatal mortality data in each month.

It is also important to note that in DHIS2 Tanzania, reported zeros are replaced with empty cells to address server lags and performance issues caused by zeros. However, this approach presents a major limitation, as it makes it difficult to differentiate between true missing values (i.e., no data reported) and ‘zero’ values (i.e., no events recorded), since both appear as blank entries in DHIS2. For the primary analysis, all blank entries were conservatively treated as missing data, acknowledging this may underestimate reporting completeness [23,30].

#### Internal consistency of reported mortality data.

For this analysis, the internal consistency of mortality data was assessed by examining the extent to which neonatal mortality patterns were similar to those observed in previous seasons. According to the WHO’s data quality guidelines, the ratio of the indicator’s value for the reference year to the average of the preceding three years should fall within ±33% [44]. The ± 33% threshold for assessing internal consistency was selected based on commonly applied data quality assessment approaches used in routine health information systems, particularly within DHIS2 evaluations and WHO data quality review frameworks [44]. We applied this threshold to compare the mean reported NMR for the 35 facilities in the most recent years (2021, 2022, and 2023) with the corresponding data for 2024. Additionally, a sensitivity analysis using a broader threshold of ±50% was conducted to evaluate the robustness of ±33% threshold.

#### External plausibility comparison.

External plausibility was assessed by comparing the consistency of reported inpatient neonatal mortality rates from DHIS2 with survey estimates from the Tanzania Demographic and Health Survey (TDHS) 2022/23 [7] and the United Nations Inter-Agency Group for Child Mortality Estimation (UN-IGME) 2024 estimates for Tanzania [2]. The 2022/23 TDHS estimate for neonatal mortality was 24/1000 live births, while the UN-IGME estimate was 21/1000 live births. It is important to note that hospital neonatal mortality rates from DHIS2 are not expected to align directly with population-based NMRs from sources such as DHS or UN-IGME, as they are calculated using different denominators or data collection methods [24]. However, quantifying these differences within Tanzanian referral hospitals will provide an understanding of data system performance that broader studies cannot offer. A sensitivity analysis was conducted to account for 20% of home births with unknown outcomes (from DHS), using the 2024 inpatient NMR from DHIS2. A supplementary table is added to show the sources of bias expected and the direction on bias expected when comparing DHIS2 and survey estimates (**S1 Table**).

### Comparison of inpatient neonatal mortality rates reported in the DHIS2 database and the NEST360 Neonatal Inpatient Database (NID) across seven hospitals during the period 2021–2024

A sub-analysis was conducted to summarise and interpret the differences in the reported inpatient NMRs between the two reporting systems, DHIS2 and NID, across seven NEST360 implementing hospitals in Tanzania [30]. The hospitals included in this analysis were three RRHs (RRH1, RRH16, RRH27) and four tertiary hospitals (tertiary3, tertiary4, tertiary5, and tertiary7). The years 2021–2024 were chosen because they mark the start of data collection for NEST360 implementation, facilitating easier comparison between the two systems. We calculated the neonatal mortality ratio by dividing the DHIS2 NMR by the NID NMR (DHIS2/NID) to assess which system was over- or underreporting the inpatient NMR. It is important to note that the NID database only captures death data for inpatient babies admitted to NCUs, excluding home births, and thus reflects hospital-based neonatal mortality. In contrast, the DHIS2 database captures neonatal deaths across all units, including those beyond the neonatal unit, and is therefore expected to report a higher number of neonatal deaths. As such, neonatal deaths in both databases were used as numerators in this analysis. The denominator for both databases was the number of livebirths, which includes babies born outside the facility but later admitted. This allowed for a direct comparison between the two systems. A ratio greater than 1 indicates higher reporting in DHIS2, while a ratio less than 1 suggests higher reporting in NID. A ratio of 1 reflects equal reporting between the two systems. A forest plot was used to present the results visually [45,46].

## Results

The DHIS2 database recorded 16,433,501 live births and 85,588 neonatal deaths over the 10 years from 2015 to 2024 across all facility levels (Table 1). There was a significant increase in the reported number of institutional live births, rising by 45.0% from 1,165,334 in 2015–2,119,145 in 2024, and in reported neonatal deaths, which increased by 58.4%, from 4,358 in 2015–10,486 in 2024. Over the same period, Tanzania’s population grew from 52.1 million in 2015 to 68.5 million people in 2024, with an annual growth rate of approximately 2.9% [40]. Regional referral and tertiary hospitals accounted for 8.7% of live births and 38.4% of neonatal deaths during this period (Table 1).

**Table 1:** Institutional live births and neonatal deaths reported by health facility level, DHIS2 Tanzania (2015-2024).

	2015	2016	2017	2018	2019	2020	2021	2022	2023	2024
**Institutional live births(N)**										
Dispensaries	464,121	466,376	511,538	666,950	721,496	768,017	717,389	739,931	808,206	861,567
Health centres	276,237	304,277	362,925	443,820	501,563	587,008	631,088	674,417	754,306	805,529
District hospitals	221,280	235,861	251,478	256,179	264,026	249,472	243,533	258,323	295,964	323,559
Regional referral hospitals	155,574	151,219	151,158	146,849	135,561	106,460	94,151	90,104	84,877	73,164
National & zonal hospitals	13,762	24,250	27,651	21,588	23,861	22,594	21,369	22,288	21,183	20,496
Others*	34,360	35,312	36,788	35,839	35,553	33,855	35,078	37,431	39,860	34,830
Total	1,165,334	1,217,295	1,341,538	1,571,225	1,682,060	1,767,406	1,742,608	1,822,494	2,004,396	2,119,145
**Institutional neonatal deaths(N)**										
Dispensaries	196	273	322	294	239	252	164	161	124	129
Health centres	293	650	826	854	931	1,965	1,681	1,810	1,272	1,350
District hospitals	1,202	1,319	2,339	2,127	1,757	2,520	2,520	2,311	2,766	2,678
Regional referral hospitals	1,580	1,615	2,450	1,906	2,352	2,459	3,138	3,953	4,708	4,386
Tertiary hospitals	123	278	446	562	405	413	853	1003	773	787
Other hospitals*	964	1,005	1,535	1,908	1,846	2,076	1,756	2,103	1,724	1,156
Total	4,358	5,140	7,918	7,651	7,530	9,685	10,112	11,341	11,367	10,486

* Others hospitals include- private for-profit hospitals, private not for-hospitals/faith-based hospitals.

This table presents data on institutional live births and neonatal deaths in Tanzania, reported at the health facility level, using DHIS2 data from 2015 to 2024.

**Fig 1:**
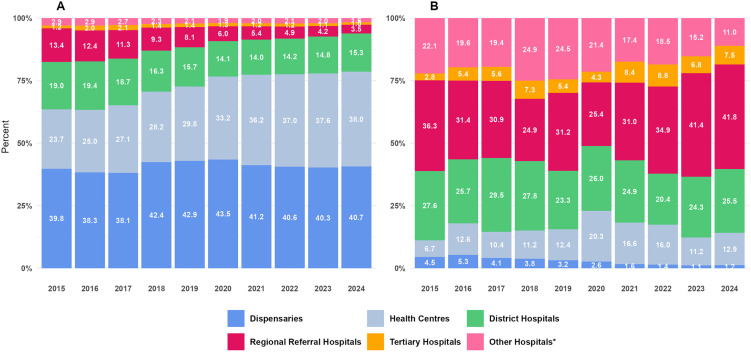
(A) Percentage of reported facility live births (N = 16,433,501) and (B) percentage of reported inpatient neonatal deaths (N = 85,588) across various health facility levels in DHIS2 Tanzania (2015-2024). *Note*: Other hospitals include- private for-profit hospitals, private not for-hospitals/faith-based hospitals. This figure illustrates the percentage of reported live births and inpatient neonatal deaths across various levels of health facilities in Tanzania, using DHIS2 data from 2015 to 2024. The color coding is as follows: Blue represents dispensaries, Grey represents health centres, Green represents district hospitals, Red represents regional referral hospitals, Brown represents zonal/national hospitals, and Pink represents other hospitals.

From 2015 to 2024, dispensaries consistently accounted for the highest percentage of live births, ranging from 38.1% to 43.5%. They were followed by health centres, which showed a steady increase in reported live births from 23.7% in 2015 to 38.0% in 2024 (Fig 1, panel A). Over the same period, regional referral hospitals reported the highest percentage of neonatal deaths compared to other facility levels, with the proportion rising from 36.3% in 2015 to 41.8% in 2024. Tertiary hospitals accounted for a smaller yet rising proportion of neonatal deaths, ranging from 4% to 11% (Fig 1, panel B).

### Quality of neonatal mortality deaths reported at various levels in Tanzania, including 28 Regional Referral Hospitals (RRHs) and seven tertiary hospitals

#### Completeness of neonatal death data.

(a) *Percentage of months in a year that a hospital reported mortality data:*

**Fig 2:**
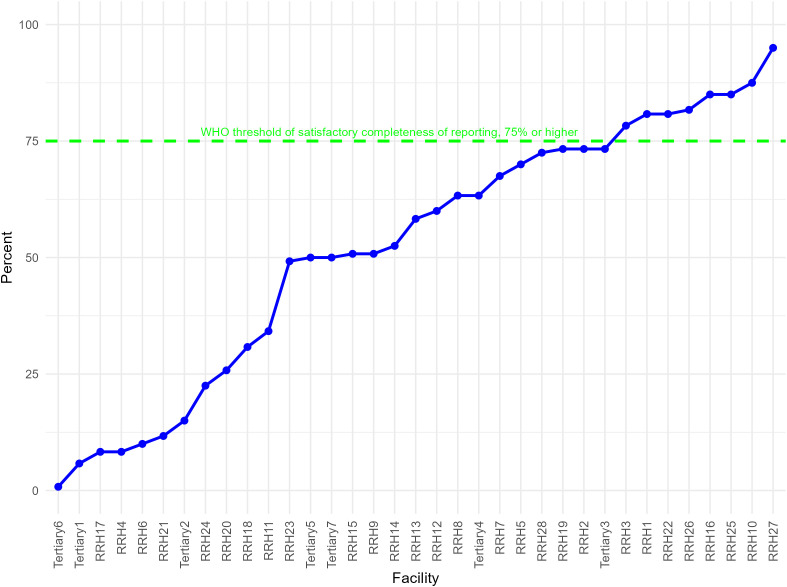
Average reporting completeness (2015-2024) among regional referral and tertiary hospitals in Tanzania using DHIS2 data (N = 35). This figure shows the average trend of reporting completeness at regional Referral and tertiary hospitals in Tanzania for ten years.

Over the 10 years, the 35 facilities achieved a median reporting completeness of 58.3% (2191 out of 4200 expected facility-months), with an interquartile range (IQR) 0.8% – 95.0%. Among the two levels analysed, RRHs reported a median of 61.7% (1881/3360 months) (IQR 8.3% – 95.0%) of the expected months, while tertiary hospitals reported a median of 50.0% (310/960 months) (IQR 0.8% – 73.3%). Reporting completeness varied across facilities: 28.5% (8/28) of RRHs reported above 75% of the expected months, and none of the tertiary hospitals reached this reporting threshold (the highest was 73.3%). Eight facilities reported completeness below 25% over the 10 years, including five regional referral hospitals and three tertiary hospitals. (Table 2, Fig 2).

**Table 2:** Monthly completeness of neonatal death reporting for Regional referral and tertiary hospitals in DHIS2, Tanzania (2015-2024) (N = 35).

Facility	Monthly contributions (N = 12)										Total months (of 120)	Percent (%)
	2015	2016	2017	2018	2019	2020	2021	2022	2023	2024		
RRH1	10	7	10	5	12	8	11	12	10	12	97	80.8
RRH2	9	12	12	11	6	7	8	3	8	12	88	73.3
RRH3	9	10	7	1	8	12	11	12	12	12	94	78.3
RRH4	2	1	1			2			3	1	10	8.3
RRH5	10		6	4	9	12	7	12	12	12	84	70.0
RRH6										12	12	10.0
RRH7	1		12	2	8	11	11	12	12	12	81	67.5
RRH8	8		9	6	2	12	7	8	12	12	76	63.3
RRH9	1	1	4		4	7	9	11	12	12	61	50.8
RRH10	11	8	12	12	12	12	12	12	7	7	105	87.5
RRH11	7	5	2			6	4	6	5	6	41	34.2
RRH12		4	12	11	5	7	8	12	9	4	72	60.0
RRH13	1	3		2	6	11	11	12	12	12	70	58.3
RRH14	9	3	6	4	8	3	3	10	11	6	63	52.5
RRH15	4			1		9	12	12	12	11	61	50.8
RRH16	4	10	10	11	10	9	12	12	12	12	102	85.0
RRH17						1	2	2	4	1	10	8.3
RRH18	1	3	4	2	2			3	11	11	37	30.8
RRH19	1	4	8	9	12	10	9	12	12	11	88	73.3
RRH20			2		4			12	11	2	31	25.8
RRH21									6	8	14	11.7
RRH22	10	12	11	6	5	9	8	12	12	12	97	80.8
RRH23	11	2	5	6			12	8	3	12	59	49.2
RRH24								6	11	10	27	22.5
RRH25	5	11	9	12	12	11	9	10	12	11	102	85.0
RRH26	10	9	10	9	10	5	11	12	11	11	98	81.7
RRH27	10	9	12	12	11	12	12	12	12	12	114	95.0
RRH28		8	10	1	8	12	12	12	12	12	87	72.5
Tertiary1									4	3	7	5.8
Tertiary2	4		1				1	8	4		18	15.0
Tertiary3	1	9	10	12	12	12	12	8	7	5	88	73.3
Tertiary4		5	12	10	5	2	8	10	12	12	76	63.3
Tertiary5						12	12	12	12	12	60	50.0
Tertiary6										1	1	0.8
Tertiary7						12	12	12	12	12	60	50.0

Tertiary- Zonal Referral Hospital/ National Hospital/Super specialist hospital.

RRH- Regional Referral Hospital.

This table shows the monthly completeness of neonatal death reporting for regional referral and tertiary hospitals in DHIS2, Tanzania, from 2015 to 2024.

Heat map color key: Red color shows completeness below 25%, Yellow color shows completeness between 25% to 50%, Orange color shows completeness between 50%−75%, Green color shows completeness above 75%.

(b) *Percentage of facilities reported deaths data monthly over time:*

The analysis showed an increase in the number of facilities reporting mortality data from 2015 to 2024. In 2015, 33.1% of the expected facility-months reported data, and by 2024, this increased to 74.5%. This reflects an improvement in the number of facilities consistently submitting data, thereby contributing to higher overall reporting completeness, rather than directly indicating the quality or accuracy of the reported data. While reporting was generally low overall across the months, there was a slight variation, with higher reporting rates observed in January (53.7%) and February (56.6%) compared to November (46.0%) and December (46.6%) (Table 3).

**Table 3:** Number of health facilities reporting deaths each month, DHIS2 Tanzania (2015-2024) (N = 35).

Year	Facility contributions (N = 35)												Total facilities (of 420)	
	Jan	Feb	Mar	April	May	June	July	Aug	Sept	Oct	Nov	Dec		Percent (%)
2015	8	8	14	14	15	12	12	13	13	11	12	7	139	33.1
2016	11	14	15	12	13	12	12	8	11	8	8	12	136	32.4
2017	20	20	14	14	20	17	17	17	16	11	16	15	197	46.9
2018	17	13	12	15	13	13	12	14	11	10	10	9	149	35.5
2019	15	16	14	17	16	18	15	14	10	13	12	11	171	40.7
2020	20	22	21	22	21	19	16	18	19	16	17	15	226	53.8
2021	22	24	25	23	20	19	22	20	19	16	18	18	246	58.6
2022	26	26	26	25	26	25	22	28	23	22	23	25	297	70.7
2023	26	30	27	26	27	29	29	27	24	25	22	25	317	75.5
2024	23	25	25	26	26	28	28	28	26	29	23	26	313	74.5
Total facilities (of 350)	188	198	193	194	197	192	185	187	172	161	161	163	2191	
Percent (%)	53.7	56.6	55.1	55.4	56.3	54.9	52.9	53.4	49.1	46.0	46.0	46.6		

This table shows the number of health facilities reporting deaths each month, using DHIS2 data from 2015 to 2024.

#### Internal consistency in reported neonatal death data.

**Fig 3:**
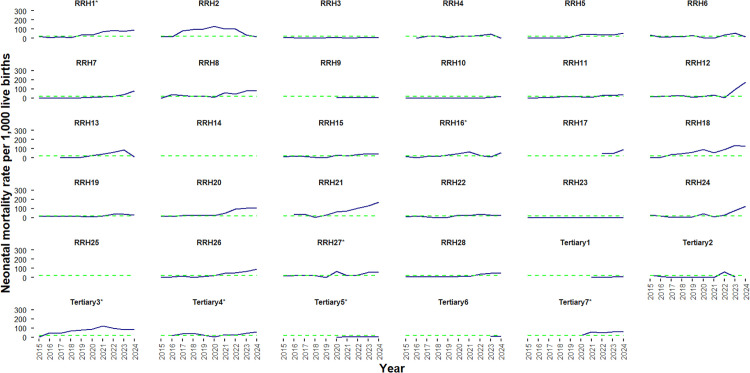
Inpatient neonatal mortality rates (NMRs) for 2015-2024: Comparison between DHIS2 and UN-IGME estimates across regional referral and tertiary hospitals in Tanzania (N = 35). *Note*: Green dotted line shows 2023 UN-IGME estimate for Tanzania (21/1000 live births); *(star) shows NEST360 implementation hospitals. This figure compares inpatient neonatal mortality rates from DHIS2 with UN-IGME estimates for regional referral and tertiary hospitals in Tanzania from 2015 to 2024.

The analysis showed that 10 of 35 hospitals (28.6%) consistently reported inpatient NMR over the past three years, defined as having a ratio of the 2024 value to the 2021–2023 mean within ±33% of the reference value. This group included seven of 28 RRHs and three of the seven tertiary hospitals (Table 4). Additionally, three facilities (8.6%) had missing data because they did not report either neonatal deaths or live births during 2021–2024, making it impossible to calculate the ratio of the 2024 value to the 2021–2023 mean. These facilities included one tertiary and two RRHs (Table 5). Several facilities showed a substantial increase in reported inpatient NMR between 2021 and 2024, with rates increasing from approximately 20–40 per 1,000 live births in earlier years to 80–200 per 1,000 live births (Fig 3). A sensitivity analysis using a wider internal consistency threshold of ±50% increased the number of facilities meeting the consistency criteria from 10 of 35 (28.6%) hospitals to 14 of 35 (40.0%). (S2 Table).

**Table 4:** Temporal consistency of inpatient neonatal mortality rate (NMR), DHIS2 Tanzania (2021-2024) (N = 35).

Facility level	Year 1(2021)	Year 2(2022)	Year 3(2023)	Year 4(2024)	Mean of Year 1-Year 3	Ratio of Year 4 to Mean of Year 1-Year 3(±33%)
RRH1	74.36	88.51	76.73	90.43	79.87	1.13
RRH2	36.43	6.56	94.98	172.95	45.99	***3.76***
RRH3	23.06	39.14	31.72	24.5	31.31	0.78
RRH4			1.36	1.36	0.45	***3.02***
RRH5	8.65	29.95	80.67	124.56	39.76	***3.13***
RRH6				28.15		
RRH7	47.44	52.99	68.7	89.4	56.38	***1.59***
RRH8	20.74	26.46	59.01	62.41	35.4	***1.76***
RRH9	12.72	35.57	46.5	48.23	31.6	***1.53***
RRH10	102.84	102.65	34.93	17.54	80.14	***0.22***
RRH11	4.74	7.59	9.58	10.25	7.3	***1.40***
RRH12	23.27	34.65	45.55	3.12	34.49	***0.09***
RRH13	46.45	35.55	38.77	58.74	40.26	***1.46***
RRH14	0.88	35.05	59.53	17.38	31.82	***0.55***
RRH15	15.38	20.38	38.33	79.86	24.7	***3.23***
RRH16	59.72	46.99	78.74	83.32	61.82	***1.35***
RRH17	3.27	7.13	10.89	4.89	7.1	0.69
RRH18		1.97	10.58	22.03	4.18	***5.27***
RRH19	11.07	29.33	30.35	41.05	23.58	***1.74***
RRH20		63.04	87.52	11.57	50.19	***0.23***
RRH21				55.08		
RRH22	22.98	37.2	46.66	42.34	35.61	1.19
RRH23	64.9	26.4	10.51	58.87	33.94	***1.73***
RRH24		48.86	44.94	91.66	31.27	***2.93***
RRH25	59.24	94.04	137.61	129.27	96.96	1.33
RRH26	17.03	39.24	38.04	30.13	31.44	0.96
RRH27	51.29	92.13	105.56	108.47	82.99	1.31
RRH28	73.14	103	131.74	170.95	102.63	***1.67***
Tertiary1	5.98	2.82	4.26	4.55	4.35	1.05
Tertiary2	0.25	61.41	8.47		23.38	
Tertiary3	121.58	100.3	83.19	83.18	101.69	0.82
Tertiary4	31.4	26.36	45.8	57.99	34.52	***1.68***
Tertiary5	7.75	3.88	5.46	8.87	5.7	***1.56***
Tertiary6			7.75	14.85	2.58	***5.76***
Tertiary7	56.98	51.43	60.81	66.46	56.41	1.18

No inpatient NMR reported.

According to WHO guidance, ratios <0.67 or >1.33 indicate reported data in DHIS2 for reference year was inconsistent with the mean of the preceding 3 years.

This table shows the temporal consistency of the inpatient neonatal mortality rate (NMR) using DHIS2 data from 2021 to 2024.

Grey shaded cells show no inpatient NMR reported.

**Table 5:** Temporal consistency of neonatal deaths and live births, DHIS2 Tanzania (2021-2024) (N = 35).

	Neonatal deaths				Live births			
Facility level	2021	2021	2021	2021	2021	2021	2021	2021
RRH1	635	674	592	532	8539	7615	7715	5883
RRH2	76	11	155	202	2086	1678	1632	1168
RRH3	216	302	256	190	9367	7715	8071	7754
RRH4			4	1	5408	6041	2932	734
RRH5	24	80	207	282	2773	2671	2566	2264
RRH6				17			468	604
RRH7	138	154	205	248	2909	2906	2984	2774
RRH8	34	40	94	87	1639	1512	1593	1394
RRH9	22	61	69	75	1730	1715	1484	1555
RRH10	116	124	38	24	1128	1208	1088	1368
RRH11	11	14	20	23	2319	1845	2087	2244
RRH12	89	125	153	9	3825	3607	3359	2884
RRH13	281	185	187	201	6049	5204	4823	3422
RRH14	5	171	249	78	5685	4879	4183	4487
RRH15	36	51	79	115	2340	2503	2061	1440
RRH16	466	346	559	488	7803	7364	7099	5857
RRH17	2	3	7	3	611	421	643	614
RRH18		8	45	97	4853	4052	4252	4404
RRH19	76	198	186	240	6866	6750	6129	5846
RRH20		139	122	13	2593	2205	1394	1124
RRH21			7	13				236
RRH22	67	101	109	99	2915	2715	2336	2338
RRH23	81	32	14	72	1248	1212	1332	1223
RRH24		15	40	67		307	890	731
RRH25	116	120	221	193	1958	1276	1606	1493
RRH26	92	144	135	86	5401	3670	3549	2854
RRH27	375	639	718	571	7312	6936	6802	5264
RRH28	180	216	237	206	2461	2097	1799	1205
Tertiary1	6	4	6	6	1003	1420	1410	1319
Tertiary2	1	338	47		4001	5504	5552	5612
Tertiary3	347	264	199	184	2854	2632	2392	2212
Tertiary4	200	155	244	296	6369	5881	5327	5104
Tertiary5	17	9	11	16	2193	2321	2015	1804
Tertiary6			1	3			129	202
Tertiary7	282	233	265	282	4949	4530	4358	4243

No death/livebirth reported.

This table shows the temporal consistency of neonatal deaths and live birthsusing DHIS2 data from 2021 to 2024.

Grey shaded cells show no death/live birth reported.

#### Plausibility comparison of DHIS2 mortality compared to 2022/23 TDHS and 2023 UN-IGME.

In Tanzania, 80% of live births occur in health facilities [2]. However, when comparing the inpatient neonatal mortality rate estimates from the three sources -DHIS2, TDHS, and UN-IGME, the estimates were not similar. A sensitivity analysis was conducted to account for 20% of outcomes from home births that are unknown, as reported by the DHS. The analysis adjusted the 2024 inpatient NMR from DHIS2 using the formula (0.2*4.9) + 4.9, where 0.2 represents the estimated proportion of uncaptured home births not captured in the inpatient data, and 4.9 is the original 2024 NMR from DHIS2. After applying this adjustment, the adjusted NMR in DHIS2 was 5.9 per 1000 live births. In comparison, the 2022/23 DHS estimate for Tanzania was 24 per 1000 live births, and the UN-IGME estimate was 21 per 1000 live births. This comparison is also shown in Fig 3.

### Inpatient NMRs reported in the DHIS2 database and NEST360 Neonatal Inpatient Database (NID) across seven hospitals during the period 2021–2024

**Fig 4:**
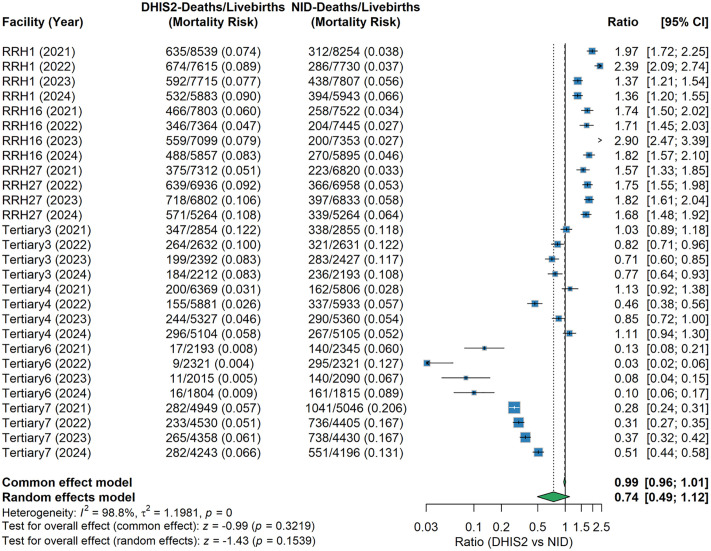
Individual analysis comparing neonatal mortality reporting between DHIS2 and NID systems across seven NEST360 hospitals, Tanzania (2021-2024) (N = 35). This figure represents an individual analysis comparing neonatal mortality reporting between the DHIS2 and NID systems across seven NEST360 hospitals in Tanzania from 2021 to 2024.

The analysis revealed that three RRHs consistently reported higher inpatient NMR in DHIS2 than in NID (Ratio > 1) throughout the five years. One tertiary hospital showed similar reporting of inpatient NMR in DHIS2 to what is captured in the NID, particularly in the later years (2023 and 2024) (Fig 4, S2 Fig).

## Discussion

This study assesses the quality of neonatal mortality data from Tanzania’s DHIS2 system in higher-level hospitals (28 regional referral hospitals and seven tertiary hospitals) from 2015 to 2024, uncovering significant underreporting and data quality issues. A direct comparison with the NEST360 NID dataset offers an opportunity for cross-validation. Between 2015 and 2024, there was a notable increase in both live births and neonatal deaths across all facility levels (**Table 1**, **Fig 1**), with regional referral hospitals and tertiary hospitals accounting for 8.7% and 38.4% of total neonatal deaths, respectively, during this period.

Variability in the completeness of neonatal mortality data was observed for both RRHs and tertiary hospitals. Some RRHs showed signs of improvement over time, particularly 28% of RRHs with higher completeness (over 75% of expected months). This same 28% of RRHs also reported higher NMRs (>100/1000 live births), suggesting that these facilities may be providing more accurate or realistic data. This trend could indicate improved data quality and reporting practices in some hospitals, although further analysis is needed to fully understand the relationship between reporting completeness and mortality accuracy [47].

Several facilities exhibited inconsistent reporting patterns, marked by significant fluctuations in reported neonatal mortality numbers and, in some cases, entire months with no data submissions. Such irregularities are particularly concerning in regional referral and tertiary hospitals, which are expected to handle high-risk newborn cases and report the highest levels of neonatal mortality [29,48–51]. Assessment of internal consistency using the WHO-recommended ±33% threshold identified substantial variability in neonatal mortality reporting across facilities. However, a sensitivity analysis using a ± 50% threshold yielded a higher proportion of hospitals meeting the consistency criteria, suggesting that some of the observed variation reflects moderate fluctuations rather than clear reporting errors.

Previous studies within Africa and LMICs have highlighted challenges with DHIS2 data [29,32,49,51–55], including maternal and newborn health indicators [21,23,24,31,35,47]. In Tanzania, neonatal mortality was underestimated by over 75%, while maternal mortality ratios and stillbirth rates are underreported by around 50% [30]. Additionally, neonatal death underreporting was a major issue in Ethiopia, with completeness rates of 45% [26]. The Multi-Country Every Newborn-Indicators Research Tracking in Hospital (EN-BIRTH) study, which assessed labor ward registers across five hospitals in Bangladesh, Nepal, and Tanzania, found that register design was a critical factor in completeness of outcome data. Excluding specific columns for discharge outcomes increases the likelihood that health care workers fail to document services and neonatal outcomes [47]. To address these issues, Tanzania is piloting a newborn register that will capture essential newborn indicators to improve monitoring of outcomes [56]. Integrating this register with DHIS2 will streamline data collection and enhance the availability of neonatal death data.

The external plausibility test revealed that the reported inpatient DHIS2 NMR for 2024 was significantly lower than survey estimates from TDHS and the UN-IGME. Specifically, DHIS2 reported a rate 75% lower than the TDHS estimate and 72% lower than the UN-IGME. This substantial underreporting indicated that policymakers relying solely on DHIS2 data would drastically underestimate the true burden, resulting in insufficient resource mobilisation and ineffective programs to achieve SDG 3.2 targets [57]. However, the literature does not clearly define the ‘plausible’ range of discrepancy between NMR estimates from facilities and surveys.

The improved neonatal mortality data reporting in DHIS2, particularly in NEST360-supported facilities, offers valuable insights into the NEST360 program’s investments, which support hospitals in strengthening care through enhanced data use [23,29,39,53,58–60]. The NEST360’s parsimonious variable list, available in the NEST360 United Nations Children’s Fund (UNICEF) Implementation Toolkit for Small and Sick Newborn Care (SSNC) [39], is an adaptable tool that enables facility and country-level comparisons to accelerate progress towards ENAP targets. Integrating this individual-level data into the government’s electronic medical records (EMRs), such as the Afya Electronic Health Management System (Afya EHMS) and the Government of Tanzania Health Operations Management Information System (GoTHOMIS), will further strengthen monitoring efforts [43,57]. Incorporating regular institutional data quality reviews and feedback into supervision is among acknowledged low-cost, effective activities for improving completeness and consistency [26,29,53,55].

Although the DHIS2 platform is widely used across Tanzania’s health system for aggregating and analysing facility data, tertiary hospitals often operate dual systems, using both DHIS2 and hospital-based electronic medical records [61]. This dual system setup can result in incomplete reporting in DHIS2, particularly when local systems lack interoperability or alignment with national reporting standards [32,62,63].

It is also worth noting that a significant proportion of neonatal deaths, ranging from 11% to 25%, are reported by ‘other’ hospitals, including private for-profit, private not non-profit, or faith-based hospitals. Many of these hospitals function as regional referral or designated district hospitals, with faith-based hospitals playing a crucial role in maternal and newborn care in rural areas, making them a valuable partner for the government. Collaborative partnerships between the public healthcare system and reporting systems are essential for improving the reporting of key indicators, such as neonatal deaths. The government’s recent adoption of a Health Information Exchange (HIE) framework offers a promising opportunity to enhance system interoperability and improve the reliability of routine health data reporting [42,64].

### Strengths and limitations

The study covers ten years and includes a large dataset comprising over 1.4 million institutional births and 34,190 neonatal deaths from 28 regional and 7 tertiary hospitals in Tanzania. This extensive dataset provides a comprehensive, comparative evaluation of the quality of inpatient neonatal mortality data across tertiary and referral hospitals. Additionally, the use of DHIS2, a widely recognized health management information system, has significant scalability potential across the broader healthcare system.

One limitation of the study is the inability to analyse neonatal mortality rates by different birthweight categories, which would offer deeper insights into selective missingness. In DHIS2, birthweight is categorised into two groups:  > 2500g and <2500g. This lack of granularity prevents a more detailed analysis of neonatal Vulnerability and selective missingness. A more precise analysis would require individual-level data on birthweight and outcomes, as seen in other studies that adjust for data quality, especially the underreporting of deaths among the smallest babies [65–67].

Another limitation is that the study is focused on higher-level hospitals, and the findings may not be representative of primary healthcare settings. This limits the generalizability of the results to the national health system. Future research should validate the observed data quality patterns and proposed interventions across a broader spectrum of facilities, including district hospitals and primary care levels, to ensure national applicability.

Additionally, DHIS2’s inability to differentiate between missing values (no data reported) and ‘zero’ values (no events captured) is a significant issue. Both are represented as blank entries, and since health facilities typically do not report ‘zero’ values, all blanks were classified as missing data. This issue has been identified in other studies in Tanzania, Kenya, and Nigeria [30,52,68].

### Implications for Health Management Information Systems (HMIS) strengthening and routine data systems in Tanzania

#### DHIS2 data validation.

Validation rules in DHIS2 should be updated to allow integer values (including ‘zero’) or default to ‘NDR’ (no data reported) for key indicator fields before submission. Additionally, Tanzania’s DHIS2 manuals and guidelines need to be revised to clearly differentiate between missing data and zero events [69,70].

#### Improving the completeness of neonatal death reporting.

The accuracy and completeness of neonatal death data can be significantly enhanced by designating a dedicated individual or healthcare workers to manage data entry at the NCU or labor ward. Additionally, providing regular supportive supervision will ensure consistent and accurate reporting [71].

#### Shift to individual-level data collection.

Transitioning from aggregate to individual-level data in EMRs is a critical step to enhance the precision and relevance of health information, particularly for monitoring neonatal outcomes. Capturing individual data, such as NEST360 neonatal inpatient data, enables more accurate tracking of key indicators, facilitating targeted interventions [39]. Moreover, developing a system that links mother and baby data in small and sick newborn care is essential for improving care delivery and outcomes [43].

#### Harmonization of death data collection systems.

Strengthening and harmonizing electronic data collection systems in Tanzania is essential. This can be achieved by integrating key stakeholders and data systems such as the Registration, Insolvency and Trusteeship Agency (RITA), Integrated Disease Surveillance and Response (IDSR), and tertiary hospitals, to create a unified approach for capturing mortality statistics [61].

#### Data-driven action.

Establish regular data review meetings at the facility, district, and regional levels to analyse trends, patterns, and anomalies [72]. These reviews should focus on identifying key areas for improvement and on ensuring that data-driven decisions lead to targeted actions to enhance health outcomes.

## Conclusion

Our study emphasizes the urgent need to improve routine facility-based neonatal mortality data collection at regional referral and tertiary hospitals in Tanzania. This can be achieved by focusing on data quality, particularly addressing the significant issue of over 75% underreporting of neonatal deaths. Furthermore, the potential reduction in DHS funding [73] after 2025 underscores the importance of strengthening routine health facility data to ensure sustained monitoring of neonatal mortality. Additionally, advanced modelling techniques, such as Bayesian frameworks, can address uncertainty and yield more precise facility-level mortality estimates.

## Supporting information

S1 Fig Tanzania health system levels and process of data collation in District Health Information System 2 Tanzania. This figure shows the organisation of the Tanzanian health system and the levels and data collation in DHIS2. *Abbreviations:* KCMC; Kilimanjaro Christian Medical Centre, CCBRT; Comprehensive Community Based Rehabilitation in Tanzania, BMC; Bugando Medical Centre, MNH; Muhimbili National Hospital, MOI; Muhimbili Orthopaedic Institute, ORCI; Ocean Road Cancer Institute, JKCI; Jakaya Kikwete Cardiac Institute. (Adapted from Shabani et al. BMC Paediatrics (2023) [https://bmcpediatr.biomedcentral.com/articles/10.1186/s12887-025-05417-x].
(TIF)

S2 Fig Reported inpatient mortality for comparing DHIS2, NID and UN-IGME estimates for NEST360 implementing facilities in Tanzania between 2021–2024. The graph depicts trends in the inpatient neonatal mortality rate (NMR) over time across various NEST360 implementing hospitals from 2021–2024, using data from three sources: the DHIS2 NEST360 neonatal inpatient dataset (NID) and UN-IGME. The lime-coloured line represents the UN estimate, while the blue and red points correspond to DHIS2 and NID data, respectively.
(TIF)

S1 Table Potential biases influencing neonatal mortality rate reporting and expected direction of effect. This table shows potential sources influencing the neonatal mortality rate and the expected direction of effect.
(DOCX)

S2 Table Temporal consistency of inpatient neonatal mortality rate (NMR), DHIS2 Tanzania (2021–2024) (N = 35) using ±50% threshold. This table shows the consistency of reporting inpatient NMR after applying a wider threshold of ±50%.
(DOCX)

S3 Table Minimum dataset. This file contains the minimum dataset used for the analysis. Sheet 1 presents the data used to generate the forest plot (2021–2024). Sheet 2 includes aggregated data on live births and neonatal deaths by health facility level for the years 2015–2024. Sheet 3 provides data on live births and neonatal deaths for each individual facility (RRH1–RRH28 and Tertiary1–Tertiary7) covering the years 2015–2024.
(XLSX)
