# Supplementary figures and images for: Counting missing babies in Tanzania: Neonatal mortality data quality from Tanzania’s District Health Information System across 28 Regional and 7 Tertiary hospitals (2015–2024)
Source: PLoS One. 2026 Jul 23;21(7):e0348874. doi: 10.1371/journal.pone.0348874 (PMC13395327; doi:10.1371/journal.pone.0348874)

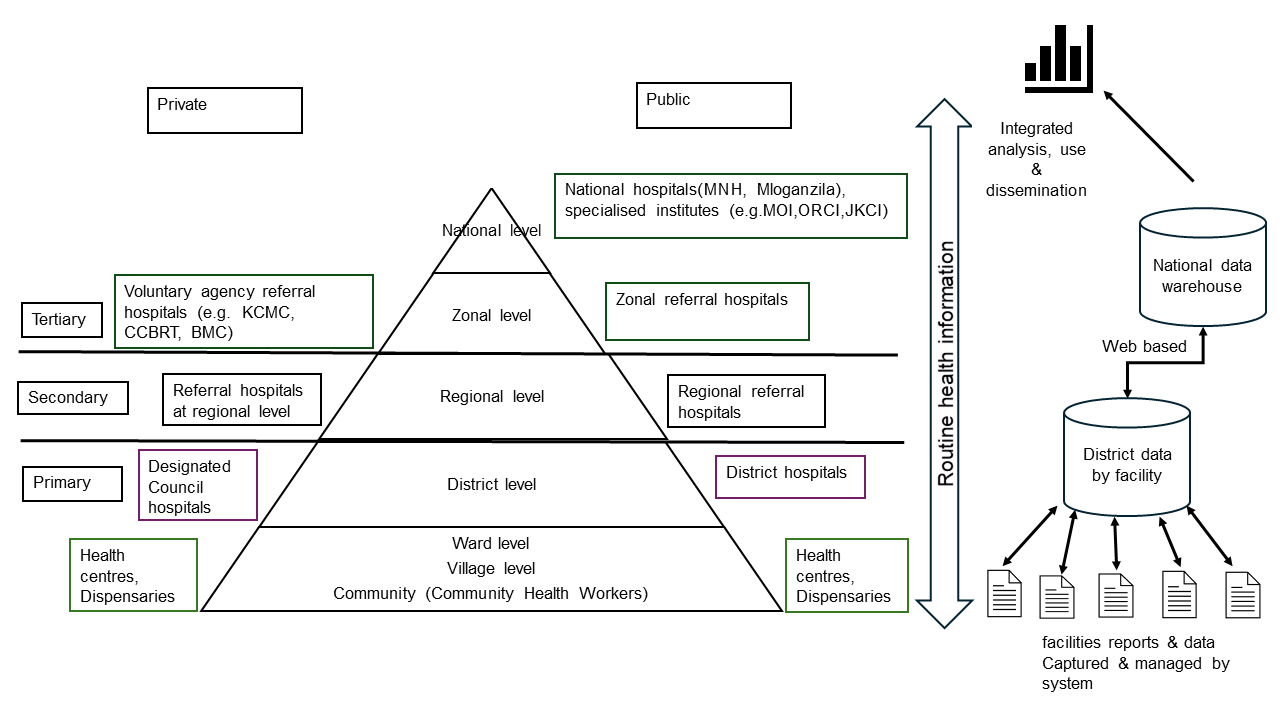

Supplement: S1 Fig — This figure shows the organisation of the Tanzanian health system and the levels and data collation in DHIS2. Abbreviations: KCMC; Kilimanjaro Christian Medical Centre, CCBRT; Comprehensive Community Based Rehabilitation in Tanzania, BMC; Bugando Medical Centre, MNH; Muhimbili National Hospital, MOI; Muhimbili Orthopaedic Institute, ORCI; Ocean Road Cancer Institute, JKCI; Jakaya Kikwete Cardiac Institute. (Adapted from Shabani et al. BMC Paediatrics (2023) [https://bmcpediatr.biomedcentral.com/articles/10.1186/s12887-025-05417-x]. (TIF) [file pone.0348874.s001.tif]

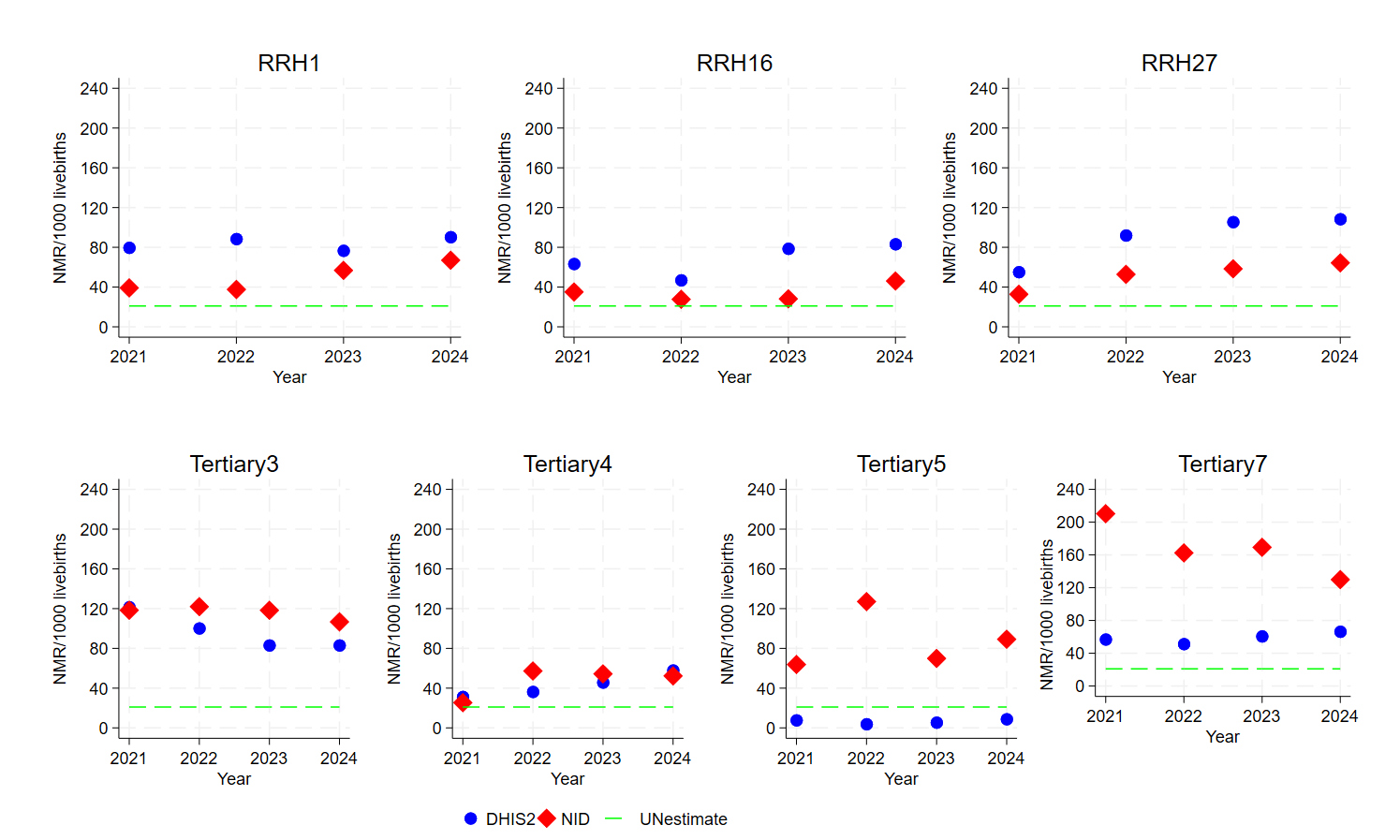

Supplement: S2 Fig — The graph depicts trends in the inpatient neonatal mortality rate (NMR) over time across various NEST360 implementing hospitals from 2021–2024, using data from three sources: the DHIS2 NEST360 neonatal inpatient dataset (NID) and UN-IGME. The lime-coloured line represents the UN estimate, while the blue and red points correspond to DHIS2 and NID data, respectively. (TIF) [file pone.0348874.s002.tif]
