# Supplementary material for: Counting missing babies in Tanzania: Neonatal mortality data quality from Tanzania’s District Health Information System across 28 Regional and 7 Tertiary hospitals (2015–2024)
Source: PLoS One. 2026 Jul 23;21(7):e0348874. doi: 10.1371/journal.pone.0348874 (PMC13395327; doi:10.1371/journal.pone.0348874)
Supplement: S1 Table — This table shows potential sources influencing the neonatal mortality rate and the expected direction of effect. (DOCX) [file pone.0348874.s003.docx]

**S1 Table: Potential biases influencing neonatal mortality rate reporting and expected direction of effect**

|  | **Likely effect on NMR estimates/expected direction of bias** | |
| --- | --- | --- |
| **Source of bias** | **DHIS2** | **DHS** |
| **Data source** |  |  |
| Different types of admin data systems,  survey data | **Decrease**: Admin data often underreports due to inconsistent reporting. | **Increase**: Surveys generally provide more comprehensive data |
| Sampling/non-sampling biases,  stochastic error from admin | **Decrease**: Errors in reporting and incomplete sampling decrease the NMR. | **Increase**: Surveys generally provide more comprehensive data. |
| **Data quality** |  |  |
| Representativeness | **Increase**: DHIS2 may have limited representativeness, especially in rural areas or smaller facilities. | **Increase**: DHS usually aims for more representative samples. |
| Classification by weight | **Increase**: DHIS2 often uses basic classifications (e.g., <2500g or >2500g), supporting detailed analysis. | **Decrease**: DHS may use more detailed weight classifications, offering more granular data. |
| **Incomplete reporting:** | **Decrease**: DHIS2 data is prone to missing or incomplete entries, leading to underreporting. | **Increase**: DHS often has more robust data verification processes, reducing missing data. |
| **Exclusion of out-of-facility deaths:** | **Decrease**: DHIS2 misses deaths that occur outside of health facilities (home births, etc.). | **Increase**: DHS typically includes both in-facility and out-of-facility deaths, providing a fuller picture. |
| **Underreporting of less severe cases:** | **Decrease**: DHIS2 may underreport less severe cases due to reporting gaps or prioritization of critical cases. | **Increase**: DHS may capture a broader range of cases, including less severe ones. |
| Exclusion of out-of-facility deaths | **Decrease**: DHIS2 misses deaths that occur outside of health facilities (home births, etc.) | **Increase**: DHS typically includes both in-facility and out-of-facility deaths, providing a fuller picture |
| Data adjustments and extrapolation based on models | **Increase**: DHIS2 may miss data corrections, leading to inflated NMR. | **Increase**: DHS applies modelling and extrapolation, which can sometimes overestimate mortality rates. |
| **Recall bias in household surveys:** | **Increase**: Not applicable to DHIS2 | **Increase**: DHS, relying on retrospective data from households, can be influenced by recall bias, especially for neonatal deaths. |
