# Supplementary material for: Counting missing babies in Tanzania: Neonatal mortality data quality from Tanzania’s District Health Information System across 28 Regional and 7 Tertiary hospitals (2015–2024)
Source: PLoS One. 2026 Jul 23;21(7):e0348874. doi: 10.1371/journal.pone.0348874 (PMC13395327; doi:10.1371/journal.pone.0348874)
Supplement: S2 Table — This table shows the consistency of reporting inpatient NMR after applying a wider threshold of ±50%. (DOCX) [file pone.0348874.s004.docx]

**S2 Table: Temporal consistency of inpatient neonatal mortality rate (NMR), DHIS2 Tanzania (2021- 2024) (N=35) using ±50% threshold**

| Facility level | Year 1(2021) | Year 2(2022) | Year 3(2023) | Year 4(2024) | Mean of Year 1-Year 3 | Ratio of Year 4 to Mean of Year 1-Year 3(±50%) |
| --- | --- | --- | --- | --- | --- | --- |
| RRH1 | 74.36 | 88.51 | 76.73 | 90.43 | 79.87 | 1.13 |
| RRH2 | 36.43 | 6.56 | 94.98 | 172.95 | 45.99 | ***3.76*** |
| RRH3 | 23.06 | 39.14 | 31.72 | 24.5 | 31.31 | 0.78 |
| RRH4 |  |  | 1.36 | 1.36 | 0.45 | ***3.02*** |
| RRH5 | 8.65 | 29.95 | 80.67 | 124.56 | 39.76 | ***3.13*** |
| RRH6 |  |  |  | 28.15 |  |  |
| RRH7 | 47.44 | 52.99 | 68.7 | 89.4 | 56.38 | ***1.59*** |
| RRH8 | 20.74 | 26.46 | 59.01 | 62.41 | 35.4 | ***1.76*** |
| RRH9 | 12.72 | 35.57 | 46.5 | 48.23 | 31.6 | ***1.53*** |
| RRH10 | 102.84 | 102.65 | 34.93 | 17.54 | 80.14 | ***0.22*** |
| RRH11 | 4.74 | 7.59 | 9.58 | 10.25 | 7.3 | 1.40 |
| RRH12 | 23.27 | 34.65 | 45.55 | 3.12 | 34.49 | ***0.09*** |
| RRH13 | 46.45 | 35.55 | 38.77 | 58.74 | 40.26 | 1.46 |
| RRH14 | 0.88 | 35.05 | 59.53 | 17.38 | 31.82 | 0.55 |
| RRH15 | 15.38 | 20.38 | 38.33 | 79.86 | 24.7 | ***3.23*** |
| RRH16 | 59.72 | 46.99 | 78.74 | 83.32 | 61.82 | 1.35 |
| RRH17 | 3.27 | 7.13 | 10.89 | 4.89 | 7.1 | 0.69 |
| RRH18 |  | 1.97 | 10.58 | 22.03 | 4.18 | ***5.27*** |
| RRH19 | 11.07 | 29.33 | 30.35 | 41.05 | 23.58 | ***1.74*** |
| RRH20 |  | 63.04 | 87.52 | 11.57 | 50.19 | ***0.23*** |
| RRH21 |  |  |  | 55.08 |  |  |
| RRH22 | 22.98 | 37.2 | 46.66 | 42.34 | 35.61 | 1.19 |
| RRH23 | 64.9 | 26.4 | 10.51 | 58.87 | 33.94 | ***1.73*** |
| RRH24 |  | 48.86 | 44.94 | 91.66 | 31.27 | ***2.93*** |
| RRH25 | 59.24 | 94.04 | 137.61 | 129.27 | 96.96 | 1.33 |
| RRH26 | 17.03 | 39.24 | 38.04 | 30.13 | 31.44 | 0.96 |
| RRH27 | 51.29 | 92.13 | 105.56 | 108.47 | 82.99 | 1.31 |
| RRH28 | 73.14 | 103 | 131.74 | 170.95 | 102.63 | ***1.67*** |
| Tertiary1 | 5.98 | 2.82 | 4.26 | 4.55 | 4.35 | 1.05 |
| Tertiary2 | 0.25 | 61.41 | 8.47 |  | 23.38 |  |
| Tertiary3 | 121.58 | 100.3 | 83.19 | 83.18 | 101.69 | 0.82 |
| Tertiary4 | 31.4 | 26.36 | 45.8 | 57.99 | 34.52 | ***1.68*** |
| Tertiary5 | 7.75 | 3.88 | 5.46 | 8.87 | 5.7 | ***1.56*** |
| Tertiary6 |  |  | 7.75 | 14.85 | 2.58 | ***5.76*** |
| Tertiary7 | 56.98 | 51.43 | 60.81 | 66.46 | 56.41 | 1.18 |
