# Supplementary material for: Counting missing babies in Tanzania: Neonatal mortality data quality from Tanzania’s District Health Information System across 28 Regional and 7 Tertiary hospitals (2015–2024)
Source: PLoS One. 2026 Jul 23;21(7):e0348874. doi: 10.1371/journal.pone.0348874 (PMC13395327; doi:10.1371/journal.pone.0348874)
Supplement: Attachment — Submitted filename: Response to reviewers.docx [file pone.0348874.s007.docx]

05/03/2026

Dear Editorial team,

***Ref: PONE-D-25-5808:* *“Counting missing babies in Tanzania: Neonatal mortality data quality from Tanzania's District Health Information System across 28 Regional and 7 Tertiary hospitals (2015-2024)”***

Thank you for the opportunity to submit a revised version of our manuscript. We are deeply appreciative to the reviewers for their thoughtful comments and suggestions. These specific comments enabled us to improve and strengthen the manuscript. Please find below our response to the reviewer’s comments.

Thank you for your consideration of this re-submission and please let us know if more information is required.

On behalf of our team,

sincerely,

*Ms Josephine M. Shabani, BSc MSc*

*Ifakara Health Institute,*

*P.O Box 78373, Dar-es-salaam, Tanzania*

*Email: jshabani@ihi.or.tz*

Table 1: specific review responses

| sn | Reviewers comments | Response |
| --- | --- | --- |
|  | Reviewer # 1 |  |
|  | General comments: Thank you for the opportunity to review this manuscript. This paper is unique and important, particularly in the current landscape of declining donor funding for large-scale surveys and the limited attention given to the use of mortality data from routine management information systems. | Thank you for reviewing the manuscript and for the positive comments regarding our manuscript and highlighting its importance and relevance. We are appreciative of your insights and thoughtful comments which have helped to improve the paper. We have provided a response to all your comments point by point as below. |
| 1 | Page 3: The manuscript reports NMR estimates from the 2010 TDHS and “2025/16 TDHS.” Please confirm whether the year 2025/16 is correct | Apologies for the typo. The year has been corrected to 2015/2016 TDHS (Line 76) |
| 2 | Page 12: The authors state that “the analysis showed improvement in the number of facilities reporting mortality data from 2015 to 2024.” The actual number of reporting facilities increased, but how do the authors conclude that reporting improved? Please clarify the basis for this interpretation~~.~~ | Thank you for your thoughtful comment. The increase in the number of facilities reporting neonatal mortality data from **2015 to 2024** was interpreted as an **improvement in reporting coverage**, rather than an improvement in the **quality or completeness** of the data.  We have revised the sentence for clarity as below (Lines 362-368)  *The analysis showed an increase in the number of facilities reporting mortality data from 2015 to 2024. In 2015,* ***33.1%*** *of the expected facility-months reported data, and by 2024, this increased to* ***74.5%.*** *This reflects an improvement in* ***reporting coverage****, rather than data quality or completeness. While reporting was generally low overall across the months, there was a slight variation, with higher reporting rates observed in January (****53.7%****) and February (****56.6%****) compared to November (****46.0%****) and December (****46.6%****) (****Table 2B****).* |
| 3 | Use of abbreviations: Abbreviation use can be strengthened throughout the paper. For example, NEST360 should be expanded when first mentioned, as well as CPAP (Page 3, Line 76). | Thank you, we have expanded all the abbreviations throughout the manuscript e.g. Newborn Essential Solutions and Technologies (NEST360), Continuous Positive Airway Pressure (CPAP), United Nations Inter-Agency Group for Child Mortality Estimation (UN-IGME) e.t.c |
| 4 | **Method section:** |  |
|  | o How did the authors access the data used in the analysis? | Thank you for this comment. We have added detail on how the data was accessed in lines 212-217:  *The aggregate DHIS2 data is available on a government portal for registered users with login credentials. The first author (JS) has access through an existing agreement between the Government and Ifakara Health Institute where JS is based. Similarly, the Neonatal Inpatient Data (NID) is available through Ifakara Health Institute which is one of the collaborating sites for NEST360 and responsible for the data collection and management. The first author is employed at Ifakara Health Institute.* |
|  | o Is this data accessible to other users/researchers? | Thank you for this comment. Yes, both datasets are accessible to other users/researchers, but access is granted according to specific procedures to ensure confidentiality and data privacy. |
|  | o In what format or template was the dataset made available or downloaded for analysis? | Thank you for this comment. DHIS2 data was downloaded in excel format and transferred to STATA for analysis. The NEST360 NID dataset was exported from REDCap in STATA format for analysis. |
| 5 | For international readers, please include a brief description of the Neonatal Inpatient Data (NID) from NEST360. What type of data or dataset does it include? | Thank you for this comment. We have provided further details on the NID from NEST360 in lines 217-220 below and added relevant references;  *NID was codesigned as parsimonious individual level dataset to be used for quality of neonatal care and includes variables covering clinical care, interventions, selected investigations and discharge outcome.* |
|  | **Results section:** |  |
| 6 | In subsection “(1) Availability of neonatal mortality data” and Table 1, the information presented does not reflect the study findings, as the analysis focuses on 35 hospitals. This content seems more appropriate for the Background section. The Results section should present findings only from the 35 hospitals to avoid confusion and ensure alignment with the study objectives. | Thank you for your helpful feedback. We understand your concern that the information in the “Availability of neonatal mortality data” subsection and Table 1 does not fully align with the study's main objectives, as it presents data from all health facilities rather than focusing on the 35 hospitals.  We agree that the Results section should primarily present findings related to the analysis of the 35 hospitals. However, we believe that the information on the overall availability of livebirth and neonatal death data across all facilities provides essential context for understanding the broader health system and effectively frames the study's findings.  In response, we have made the following changes:   - Reframed the “Availability of neonatal mortality data” section to serve as background context. This section will be positioned at the beginning of the Results section, but will no longer be linked directly to Objective 1. - The main findings of the study will now focus specifically on the 35 hospitals, including the analysis of completeness of neonatal death data, internal consistency in reported neonatal death data, and the plausibility comparison of DHIS2 mortality with the 2022/23 TDHS and 2023 UN-IGME data. - Table 1 and Figure 1 will be retained, but they will now serve as contextual data, not part of the primary results. |
| 7 | Page 12, line 272. Does an increase in the number of reported neonatal deaths necessarily indicate improvement? Please clarify the interpretation and its implications. | Many thanks for this comment. We have addressed a similar issue under comment #2. |
| 8 | The manuscript is heavily data-focused without adequate programmatic context, which may limit its readability for program managers or practitioners. Consider adding: o An overview of neonatal programs in Tanzania; o Data quality improvement activities implemented (e.g., service provider or medical recorder training, routine data quality assessments);   - Any existing MPDSR or neonatal mortality surveillance system | Thanks so much for this comment, we have added a paragraph on introduction section on;  Overview of neonatal programs in Tanzania (Lines 101-111) as below;  *Over the past two decades, Tanzania has achieved considerable reductions in child mortality, alongside recent notable declines in maternal mortality [1], prompting a strategic shift toward national and subnational interventions focused on reducing neonatal mortality [2]. During the late Millennium Development Goals (MDG) era, the increase in facility births led to the national scale-up of neonatal resuscitation programs, such as Helping Babies Breathe Additionally, the WHO's Integrated Management of Childhood Illness (IMCI) framework, which standardizes the management and prevention of childhood illnesses, was expanded to include a newborn component. Since 2010, KMC has also been scaled up nationally and incorporated into many district health budgets[3]. Despite these advancements, there remains a recognized gap for a more comprehensive small and sick newborn care package. The government has developed norms for this package and is now committed to scaling it nationally[4]. .*    Data quality improvement activities implemented (Lines 96-100)  *Common data quality issues, such as incompleteness and inconsistency, have been identified across various DHIS2 studies in Tanzania [5-9]. However, several data quality initiatives including supportive supervision, data quality assessments (DQA) and data use workshops have been implemented at various levels[7, 10].*  The Maternal and Perinatal Death Surveillance and Response (MPDSR) system is also used in Tanzania to conduct mortality audits for accountability and to identify the underlying causes of deaths. MPDSR collects data at the individual case level, whereas DHIS2 captures aggregated facility-level data. As a result, direct linkage between MPDSR and DHIS2 data is not feasible due to the differences in data structure and level of reporting. |
|  | **Discussion:** |  |
| 9 | The discussion can be strengthened by adding contextual information, such as comparisons with other countries—preferably within Africa or other LMICs. If no comparable studies exist, that should be stated as an important discussion point. Additionally, how do neonatal mortality data compare with maternal or other mortality indicators reported in DHIS2?  What is known about the overall quality of DHIS2 data in Tanzania? Strengthening routine data systems is crucial, especially given current reductions in donor-funded programs such as DHS.  Based on the analysis, what specific recommendations can the authors offer for improving data quality? | Thank you for this constructive comment. We have strengthened the Discussion section by incorporating additional contextual information and references on the overall quality of DHIS2 data in Tanzania, including comparisons with maternal and child health indicators in terms of data completeness and quality. The Discussion has also been expanded to include evidence from other African and LMIC settings. Furthermore, we have added a new subsection on the implications for strengthening HMIS and routine data systems, outlining specific recommendations to improve routine data coverage, reporting quality, and neonatal mortality monitoring in Tanzania. |
|  | **Limitations**: |  |
| 10 | The limitations section should be revisited. The analysis could potentially have been disaggregated by public versus private hospitals. Are there additional limitations related to the use of historical data or known data quality issues? | Thank you for your comment. We appreciate your suggestion to consider private hospitals. In Tanzania, 84.9% of healthcare facilities are public, while the remaining 15.1% are private. Of these private facilities, 62.2% are Private For Profit (PFP) and 37.8% are Private Not for Profit (PNPF) or faith-based hospitals. Given this distribution, it is expected that a higher number of neonatal deaths would be reported from PFP hospitals. However, this analysis focused on 35 high-level facilities, consisting of 28 regional referral hospitals (RRH) and 7 tertiary hospitals, all of which are public.  Again, thank you for highlighting the issue of historical data. This study extracted data from 2004 to 2024, but for the analysis, we prioritized more recent and complete data(2015-2024), as older data is likely to be more biased. |
|  | Reviewer # 2 |  |
|  | Thank you for involving me in the peer review of this manuscript. I have provided the authors with the detailed review report below and invite authors to address all the points raised during my review. Regards. | Thank you for taking the time to thoughtfully review our manuscript. We deeply appreciate your insightful comments and constructive feedback, which have been invaluable in enhancing the quality of the paper. We particularly appreciate how you framed your review by clearly outlining the *issues, their significance, and your suggestions for improvement*. We have carefully addressed each of your comments in detail in the following response. |
|  | **Overall assessment** This is a timely, policy-relevant, and important manuscript addressing a critical gap in neonatal mortality surveillance in Tanzania. The focus on secondary and tertiary hospitals, the long-time span (2015–2024), and the triangulation across DHIS2, DHS, UN-IGME, and NEST360 NID are major strengths. The paper convincingly demonstrates substantial under-capture and inconsistency in DHIS2 neonatal mortality reporting.  However, the manuscript would benefit from clearer conceptual framing, stronger justification of methodological choices, more cautious interpretation of comparisons, and clearer articulation of implications for HMIS strengthening. Several areas also require clarification to avoid misinterpretation by readers unfamiliar with DHIS2 or inpatient vs population-based mortality metrics. | Thank you for your positive and constructive feedback on our manuscript. We greatly appreciate your recognition of the timeliness, policy relevance, and strengths of the study, especially in addressing the critical gap in neonatal mortality surveillance in Tanzania.  In response to your feedback, we have revised several sections of the manuscript, including:   - Expanding and clarifying the connection between the previous study and the current one. The earlier study evaluated the quality and availability of neonatal data in DHIS2 Tanzania and found that the neonatal mortality rate (NMR) was underestimated by over 75%.[3] The current study builds on these findings by focusing on regional referral and tertiary hospitals, where most neonatal deaths are reported, providing a deeper understanding of data quality issues at higher-level healthcare facilities. - Adding statistical validation to ensure the consistency of the comparison between the mean NMR for 2021–2023 and 2024, using a WHO-guided ±33% threshold. A sensitivity analysis with a ±50% threshold has also been included to further strengthen the robustness of the assessment. - Specifying the exact numerator and denominator used to calculate the DHS NMR, DHIS2 NMR, and NEST360 NID NMR, ensuring full transparency in the methodology and analytical rigor. - Expanding the limitations section to highlight an important unacknowledged limitation: the findings from this small subset of high-level facilities may not be generalizable to the thousands of lower-level facilities, which limits the external validity of the study.   You will find updates throughout the manuscript. |
|  | **Major comments** |  |
| 1 | Clarify the conceptual distinction between inpatient neonatal mortality and population-based neonatal mortality  **Issue:**  Throughout the manuscript, inpatient NMRs from DHIS2 are repeatedly compared to DHS and UN-IGME population-based NMRs. While the authors acknowledge this difference, the conceptual implications are not sufficiently foregrounded.  **Why this matter:**  Readers may incorrectly interpret discrepancies as purely data quality problems, rather than also reflecting fundamentally different denominators, case-mix, and risk profiles.  **Suggestions:**  Explicitly state early (Introduction or Methods) that: DHIS2 hospital NMRs are not expected to match population-based NMRs. Referral hospitals should, if anything, have higher inpatient mortality, not lower. Consider adding a conceptual figure or box explaining: Expected direction of bias when comparing DHIS2 vs DHS/UN estimates. Strengthen the argument that the direction and magnitude of discrepancy strongly indicate underreporting, even after accounting for these differences. | Thank you for your comment. We have updated the methodology section under Data Sources to clarify the differences between population-based data and inpatient neonatal mortality. The revised text can be found in lines 145-149 as follows:  *For this analysis, national neonatal mortality rate refers to deaths in the first 28 days after birth over 1000 live births captured by national health surveys like DHS. Inpatient neonatal mortality rate is the official DHIS2 and WHO indicator. It refers to the deaths of neonates during admission (so this may not include to 28 days) amongst those admitted to health care facilities[11].*  We have also added a paragraph on the external plausibility of the reported mortality data (Lines 277-279), as follows:  *It is important to note that hospital neonatal mortality rates from DHIS2 are not expected to align directly with population-based NMRs from sources such as DHS or UN-IGME, as they are calculated using different denominators or data collection methods [12]*  Moreover, we have added a Table in Supplementary materials (S2 Table) explaining sources of bias expected direction of bias when comparing DHIS2 vs DHS/UN estimates. |
| 2 | Justify the choice of ±33% threshold for internal consistency more explicitly  **Issue:**  The ±33% WHO threshold is applied, but the rationale and appropriateness for neonatal mortality in referral hospitals is not fully discussed.  **Why this matter:**  Given the high year-to-year variability expected in hospital case-mix, readers may question whether ±33% is too strict or too lenient.  **Suggestions:**  Provide a short justification explaining why this threshold is appropriate for: High-volume referral hospitals Mortality indicators rather than service-delivery indicators Consider a sensitivity analysis using a wider threshold (e.g. ±50%) or explicitly acknowledge this as a limitation. | Thank you for this important comment. The ±33% threshold was selected based on WHO Routine Health Information System data quality assessment guidelines and commonly applied DHIS2 evaluation approaches.[13].  To further assess its appropriateness for neonatal mortality in referral hospitals, we conducted a sensitivity analysis using a wider ±50% threshold. This increased the number of facilities meeting consistency criteria from 10(28.5%) to 14(40.0%) out of 35 hospitals, indicating that some facilities exhibited moderate variability rather than true reporting inconsistency. These findings suggest that assessments of internal consistency are sensitive to threshold selection, particularly in referral settings where fluctuations in neonatal mortality may reflect variations in case severity and referral patterns. The rationale and implications have now been clarified in the revised manuscript.  We have revised methodology, results and discussion sections for incorporating the updates, can be found in following lines ;(263 – 265), (379-390) and (450-454) respectively.  *Methods: The ±33% threshold for assessing internal consistency was selected based on commonly applied data quality assessment approaches used in routine health information systems, particularly within DHIS2 evaluations and WHO data quality review frameworks[13].*  *Results:* *The analysis showed that 10 of 35 hospitals (28.6%) consistently reported inpatient NMR over the past three years, defined as having a ratio of the 2024 value to the mean of 2021-2023 within ±33% of the reference value. This group included seven of 28 RRHs and three of the seven tertiary hospitals (****Table 3******B****). Additionally, three facilities (8.6%) had missing data, because did not report either neonatal deaths or live births during 2021-2024, making it impossible to calculate ratio of the 2024 value to the 2021-2023 mean. These facilities included one tertiary and two RRHs (****Table 3A****). Several facilities showed a substantial increase in reported inpatient NMR between 2021 and 2024, with rates increasing from approximately 20–40 per 1,000 live births in earlier years to 80–200 per 1,000 live births (****Fig 3****). A sensitivity analysis using a wider internal consistency threshold of ±50% increased the number of facilities meeting the consistency criteria from 10 of 35 (28.6%) hospitals to 14 of 35 (40.0%). (****S1 Table****).*  *Discussion: Assessment of internal consistency using the WHO-recommended ±33% threshold identified substantial variability in neonatal mortality reporting across facilities. However, sensitivity analysis applying a wider ±50% threshold resulted in a higher proportion of hospitals meeting consistency criteria, indicating that part of the observed variation reflects moderate fluctuations rather than clear reporting errors.* |
| 3 | Interpretation of very high inpatient NMRs needs strengthening  **Issue:**  Some hospitals report inpatient NMRs exceeding 100 per 1,000 live births, which is briefly noted but not deeply explored. **Why this matter:** Such values could reflect: Severe referral bias Denominator problems (e.g. births counted outside neonatal units) Data entry or classification errors  **Suggestions:**  Expand the Discussion to explicitly address: Whether these extreme values are plausible Whether they suggest numerator inflation, denominator undercounting, or both Clarify whether live births used as denominators include: Only births occurring in the hospital Or also referrals admitted post-birth | Thank you very much also alerting to the issue **very high inpatient neonatal mortality rates (NMRs) above 100** which may be related to denominator missingness, data entry errors or to true variation in case mix with a much higher risk population.  Our study intentionally focuses on **high-level facilities**, such as **Regional Referral Hospitals (RRHs)** and **Tertiary hospitals,** which handle a mix of **cases** and **severe conditions**, often functioning as **referral hospitals**. As a result, we would expect these facilities to report **higher NMRs** compared to lower-level facilities and compared the national NMR. While **higher NMRs** may suggest that these hospitals are providing more **accurate** or **realistic data,** further analysis is required to better understand the validity of the highest **reported NMRs.**  In the analysis we found that either the **numerator** (neonatal deaths) or the **denominator** (livebirths) exhibited fluctuations . For example, **RRH5** reported a sharp decrease in **livebirths** in 2024, while **neonatal deaths** remained stable, leading to a significant increase in the reported **NMR**.However, we are not sure if these data shifts are caused by data quality issues or **tallying system** or **human errors** during **data entry.** It is important to note that the **livebirths** reported in **DHIS2** include both **inborns** (births occurring at the facility) and **outborns** (referrals admitted post-birth), which may affect the accuracy of the reported **NMRs**.  We have added a paragraph in the **Discussion section** in **Lines 438-444**, as outlined below:  *Some RRHs showed signs of improvement over time particularly 28****% of RRHs with higher completeness (over 75% of expected months). This same 28% of RRHs*** *also reported* ***higher NMRs (>100/1000 live births)****, suggesting that these facilities may be providing more accurate or realistic data. This trend could indicate improved data quality and reporting practices in some hospitals, although further analysis is needed to fully understand the relationship between* ***reporting completeness*** *and mortality* ***accuracy[14]***. |
| 4 | Completeness analysis would benefit from clearer interpretation  **Issue:**  The manuscript reports completeness rigorously but stops short of interpreting what level of completeness is operationally acceptable for mortality surveillance.  **Suggestions:**  Explicitly state why >80% completeness was chosen as a benchmark. Discuss implications of <60% completeness for: Trend analysis Facility benchmarking National aggregation Consider classifying facilities into performance tiers (e.g. high, moderate, low completeness) and summarizing their characteristics. | Thank you for your insightful comment. We have revised the part on data completeness analysis and introduced the following cut-off points (<25%, 25–50%, 50–75%, and >=75%) to assess the extent of complete reporting. Facilities with a completeness rate of 75% or higher are considered to have high reporting. The 75% cut-off point is based on the WHO data quality framework[13]. For DHIS2 Tanzania, the district's reporting threshold for various monthly forms (such as delivery and postnatal care forms) is set at 90%.  We have updated Table 2A to reflect the completeness of reporting threshold for each facility and have added **Fig 2** to show the trend analysis for 2024 reporting, including the 75% reference line. |
| 5 | Strengthen the policy and systems implications |  |
|  | **Issue:**  The Conclusions and Discussion point to “improving reporting” but remain relatively general.  **Suggestions:**  Be more explicit about actionable system-level recommendations, such as: Linking DHIS2 neonatal death reporting to routine mortality audit processes Harmonising DHIS2 indicators with NEST360/NID definitions Introducing automated plausibility checks within DHIS2 Clarify how high-performing RRHs could be used as: Learning sites Mentorship hubs Sentinel surveillance facilities Minor comments and technical suggestions Terminology Use “live births” consistently (avoid alternating with “livebirths”). Clarify when “neonatal mortality rate” refers specifically to inpatient mortality. Grammar and clarity Several sentences are long and could be tightened, particularly in the Introduction and Discussion. Example: “Although the percentages of births in health facilities has increased…” → should be “have increased”. | Thank you very much for this valuable comment. We have responded to this comment at the overall assessment comment and add the section of “Implications for HMIS strengthening and routine data systems in Tanzania” under the discussion section in the following lines (571-596).  We have also revised improve grammar and clarity, particularly in the discussion and introduction sections, based on your suggestions. |
| 6 | Figures |  |
|  | Ensure all figures clearly label: Denominators Facility types Data sources | Thank you for this comment. I have revised figure labels as follows:  *Fig 1. (A) Percentage of reported facility livebirths (N=16,433,501) and (B) percentage of reported inpatient neonatal deaths (N=85,588) across various health facility levels in DHIS2 Tanzania (2015-2024).*  *Fig 2.* Average reporting completeness over ten years in regional referral and tertiary hospitals in Tanzania using DHIS2 data (N=35).  *Fig 3. Inpatient neonatal mortality rates (NMRs) for 2015-2024: Comparison between DHIS2 and UN-IGME estimates across regional referral, zonal, and national hospitals in Tanzania (N=35).*  *Fig 4.* Individual analysis comparing neonatal mortality reporting between DHIS2 and NID systems across seven NEST360 hospitals, Tanzania (2021-2024) (N=7). |
|  | Tables |  |
|  | Table 3 is very dense; consider splitting into: Deaths & births NMR & consistency metrics | Thank you for your helpful comment. We have revised the table as suggested and now present the data in two separate tables: **Table 3a**, which shows livebirths and neonatal deaths, and **Table 3b**, which displays inpatient neonatal mortality rates and consistency metrics. |
| 7 | Methods transparency |  |
|  | Specify whether zero values in DHIS2 were treated as: True zero deaths Or missing reports Clarify how missing live birth data were handled in NMR calculations. | Thank you for this comment. The DHIS2 team in Tanzania replaced reported zeros with empty cells in the system to address server lags and performance issues caused by zeros. However, they acknowledge that this approach presents a major limitation, as it makes it difficult to differentiate between true missing values (i.e., no data reported/missing reports) and ‘zero’ values (i.e., no events recorded/true zero deaths), since both appear as blank entries in DHIS2.  Given the wide range of services provided at higher-level facilities, such as Regional Referral Hospitals (RRHs) or Tertiary-level hospitals, it is unlikely that no neonatal deaths or livebirths occurred over a one-year period. Therefore, in this study, any missing data entries for livebirths or neonatal deaths were classified as "missing" (i.e., no data reported). As a result, the observed completeness of reporting likely underrepresents the true situation.  This issue of ‘zero’ reporting has been noted in other studies, including: the assessment of completeness in malaria indicator data reporting via the DHIS2 system in Kenya[15], the evaluation of the quality of routine facility data for monitoring priority maternal and newborn indicators in Gombe State, Nigeria[16] , and the evaluation of availability and quality of selected Every Newborn Action Plan (ENAP) indicators in Tanzania from 2015‑2022[9].  The revised paragraph under *methods* can be found in lines 250-255 as follows:  *It is also important to note that,* *in DHIS2 Tanzania, the reported zeros are replaced with empty cells in the system to address server lags and performance issues caused by zeros. However, this approach presents a major limitation, as it makes it difficult to differentiate between true missing values (i.e., no data reported) and ‘zero’ values (i.e., no events recorded), since both appear as blank entries in DHIS2. For the primary analysis, all blank entries were conservatively treated as missing data, acknowledging this may underestimate reporting completeness*.    We have also rephrased the *limitations* part in Lines 576-580 as follows:  *Additionally, DHIS2's inability to differentiate between missing values (no data reported) and ‘zero’ values (no events captured) is a significant issue. Both are represented as blank entries, and since health facilities typically do not report ‘zero’ values, all blanks were classified as missing data. This issue has been identified in other studies in Tanzania, Kenya and Nigeria[9, 15, 16].*  We have also rephrased the” Implications for HMIS strengthening and routine data systems in Tanzania” part in Lines 584-587 as follows:  *DHIS2 data validation: Validation rules in DHIS2 should be updated to allow integer values (including ‘zero’) or default to 'NDR' (no data reported) for key indicator fields before submission. Additionally, Tanzania's DHIS2 manuals and guidelines need to be revised to clearly differentiate missing data and zero events[10, 17]* |
| 8 | Ethics statement |  |
|  | Well written and appropriate; no major concerns. Specific questions for the authors (points needing clarification) | Thank you for your positive feedback on the ethics statement. As you noted, there were no major concerns, and we have addressed the issue by moving the **ethics statement** to the **Methodology section**, as per the journal’s guidelines. |
| 9 | Denominator definition: |  |
|  | Are live births in DHIS2 limited to births occurring within the facility, or do they include births occurring elsewhere but later admitted? | Many thanks for this comment. The livebirths reported in each facility in DHIS2 comprises of both inborns and outborns i.e. births occurring elsewhere but later admitted.  We have also revised the paragraph under *methods* in lines 304-306 as follows;  *The denominator for both databases was the number of livebirths, which includes babies born outside the facility but later admitted. This allowed for a direct comparison between the two systems.* |
| 10 | Zero reporting: |  |
|  | How did you distinguish between true zero neonatal deaths and months with missing reports recorded as zero? | Many thanks for this comment. We have addressed a similar issue under comment #7. |
| 11 | Referral bias: |  |
|  | Did you explore whether hospitals with higher completeness also had higher reported NMRs, suggesting more realistic reporting? | Thank you for your thoughtful comment. We explored the relationship between **reporting completeness** and **reported neonatal mortality rates (NMRs)** across the facilities. Our analysis revealed that **28.5% of the facilities with higher completeness** (over 75%) also reported **higher NMRs**, which may indicate that these facilities are providing more accurate or realistic data. We have clarified this finding in the manuscript and expanded on its implications in the **Discussion section**, where we highlight the need for further research to explore the factors influencing these trends. Please refer to lines **438-444** for the updated discussion, as shown below:  *Some RRHs showed signs of improvement over time particularly 28****% of RRHs with higher completeness (over 75% of expected months). This same 28% of RRHs*** *also reported* ***higher NMRs (>100/1000 live births),*** *suggesting that these facilities may be providing more accurate or realistic data. This trend could indicate improved data quality and reporting practices in some hospitals, although further analysis is needed to fully understand the relationship between* ***reporting completeness*** *and mortality* ***accuracy[14].*** |
| 12 | NEST360 comparison: |  |
|  | Were case definitions for neonatal death fully harmonised between DHIS2 and NID, particularly regarding timing (early vs late neonatal death)? | Thank you for your valuable comment. In this analysis, we did not conduct a disaggregated assessment based on the timing of neonatal deaths (early versus late neonatal deaths). It is important to note that both DHIS2 and NID capture predischarge neonatal deaths, which differ from the standard definition of all neonatal deaths occurring within the first 28 days of life.  Although the analysis did not differentiate between early neonatal deaths (within the first 7 days of life) and late neonatal deaths (between 7 and 28 days), we acknowledge that such disaggregation could provide additional insights and should be considered in future analyses. Given that approximately 75% of neonatal deaths occur within the first week of life, and the average length of hospital stay is about six days—combined with the fact that nearly 85% of births occur in health facilities—it is likely that most neonatal deaths are captured before discharge.  For this study, we focused on neonatal death as a whole, consistent with the definitions used in both data sources. |
| 13 | Private sector role: |  |
|  | Given the “Others” category contributes substantially to deaths, what are the implications for national neonatal mortality surveillance beyond public hospitals? | Thank you for your insightful comment. We acknowledge that the **"Others"** category, contributes a substantial portion of neonatal deaths in the data. In this analysis “Others” reflects deaths occurring in **non-public healthcare settings** such as **private not for hospitals includinh** Faith Based hospitals(PNFP) or **Private for Profit(PFP).** To address this, we have expanded the discussion section by adding the following paragraph (lines 494-503) regarding **"Other"** facilities:  *It is also worth noting that a significant proportion of neonatal deaths, ranging from 11% to 25%, are reported by ‘other’ hospitals, including private for-profit, private not for profit or faith-based hospitals. Many of these hospitals function as regional referral or designated district hospitals, with faith-based hospitals playing a crucial role in maternal and newborn care in rural areas, making them a valuable partner for the government. Collaborative partnerships between the public healthcare system and reporting systems are essential for improving the reporting of key indicators, such as neonatal deaths. The government’s recent adoption of a Health Information Exchange (HIE) framework offers a promising opportunity to enhance system interoperability and improve the reliability of routine health data reporting[18, 19].* |
| 14 | Overall recommendation:  This manuscript addresses a critical gap in neonatal mortality measurement and has strong potential to influence health information system strengthening in Tanzania and similar settings. With clearer framing, strengthened interpretation, and more explicit policy implications, it would make a valuable contribution to the literature. | Thank you again for taking the time to review our manuscript. We greatly appreciate your valuable insights and thoughtful feedback, which have significantly enhanced the quality of the paper. |
|  | Academic editor | Response |
| 1 | Please ensure that your manuscript meets PLOS ONE's style requirements, including those for file naming. The PLOS ONE style templates can be found at  https://journals.plos.org/plosone/s/file?id=wjVg/PLOSOne_formatting_sample_main_body.pdf and  <https://journals.plos.org/plosone/s/file?id=ba62/PLOSOne_formatting_sample_title_authors_affiliations.pdf>. | Thank you very much. We have revised the manuscript to align with PLOS ONE’s style guidelines. |
| 2 | We note that the grant information you provided in the ‘Funding Information’ and ‘Financial Disclosure’ sections do not match.  When you resubmit, please ensure that you provide the correct grant numbers for the awards you received for your study in the ‘Funding Information’ section. | Thanks so much for this comment. This work is funded through the NEST360 Alliance with thanks to the John D. and Catherine T. MacArthur Foundation, the Bill & Melinda Gates Foundation, ELMA Philanthropies, The Children’s Investment Fund Foundation UK, The Lemelson Foundation, The Sall Family Foundation, and the Ting Tsung and Wei Fong Chao Foundation under agreements to William Marsh Rice University under the following subaward Number X03129551 |
| 3 | We noted in your submission details that a portion of your manuscript may have been presented or published elsewhere. [We have one figure- S2 Fig. Health system and process of data collation in District Health Information System 2 Tanzania. This figure has been adapted from Shabani et.al BMC Paediatrics (2023) https://bmcpediatr.biomedcentral.com/articles/10.1186/s12887-025-05417-x] Please clarify whether this [conference proceeding or publication] was peer-reviewed and formally published. If this work was previously peer-reviewed and published, in the cover letter please provide the reason that this work does not constitute dual publication and should be included in the current manuscript. | Thank you for your thoughtful comment. We would like to confirm that **S1 Fig. Health system and process of data collation in District Health Information System 2 Tanzania** was previously published in our peer-reviewed article **Shabani et al., BMC Paediatrics (2023)**. [https://bmcpediatr.biomedcentral.com/articles/10.1186/s12887-025-05417-x]. The figure in that paper was used to illustrate similar concepts, but in this manuscript, we have adapted it for the purpose of providing a clearer understanding of the **health system in Tanzania** and **data collation in DHIS2.** We believe that reusing this figure does not constitute **dual publication**, as it has been **modified** to better align with the specific focus of this study. The figure is crucial for explaining the context and processes within the Tanzanian health system, which is central to the current manuscript. We have appropriately cited the original publication to acknowledge the source of the figure and its prior use. We hope this clarification resolves any concerns. |
| 4 | Your ethics statement should only appear in the Methods section of your manuscript. If your ethics statement is written in any section besides the Methods, please move it to the Methods section and delete it from any other section. Please ensure that your ethics statement is included in your manuscript, as the ethics statement entered into the online submission form will not be published alongside your manuscript. | Thank you for your helpful comment. We have moved the ethics statement to the **Methods** section, as per your suggestion. The ethics statement has now been removed from any other sections of the manuscript to ensure compliance with the journal's guidelines. |
| 5 | We note that Figure S1 in your submission contain [map/satellite] images which may be copyrighted. All PLOS content is published under the Creative Commons Attribution License (CC BY 4.0), which means that the manuscript, images, and Supporting Information files will be freely available online, and any third party is permitted to access, download, copy, distribute, and use these materials in any way, even commercially, with proper attribution. For these reasons, we cannot publish previously copyrighted maps or satellite images created using proprietary data, such as Google software (Google Maps, Street View, and Earth). For more information, see our copyright guidelines: <http://journals.plos.org/plosone/s/licenses-and-copyright>.  We require you to either (1) present written permission from the copyright holder to publish these figures specifically under the CC BY 4.0 license, or (2) remove the figures from your submission:   a. You may seek permission from the original copyright holder of Figure S1 to publish the content specifically under the CC BY 4.0 license.  We recommend that you contact the original copyright holder with the Content Permission Form (http://journals.plos.org/plosone/s/file?id=7c09/content-permission-form.pdf) and the following text:  “I request permission for the open-access journal PLOS ONE to publish XXX under the Creative Commons Attribution License (CCAL) CC BY 4.0 (http://creativecommons.org/licenses/by/4.0/). Please be aware that this license allows unrestricted use and distribution, even commercially, by third parties. Please reply and provide explicit written permission to publish XXX under a CC BY license and complete the attached form.”  Please upload the completed Content Permission Form or other proof of granted permissions as an "Other" file with your submission.  In the figure caption of the copyrighted figure, please include the following text: “Reprinted from [ref] under a CC BY license, with permission from [name of publisher], original copyright [original copyright year].”  b. If you are unable to obtain permission from the original copyright holder to publish these figures under the CC BY 4.0 license or if the copyright holder’s requirements are incompatible with the CC BY 4.0 license, please either i) remove the figure or ii) supply a replacement figure that complies with the CC BY 4.0 license. Please check copyright information on all replacement figures and update the figure caption with source information. If applicable, please specify in the figure caption text when a figure is similar but not identical to the original image and is therefore for illustrative purposes only.  The following resources for replacing copyrighted map figures may be helpful:  USGS National Map Viewer (public domain): http://viewer.nationalmap.gov/viewer/  The Gateway to Astronaut Photography of Earth (public domain): http://eol.jsc.nasa.gov/sseop/clickmap/  Maps at the CIA (public domain): https://www.cia.gov/library/publications/the-world-factbook/index.html and https://www.cia.gov/library/publications/cia-maps-publications/index.html  NASA Earth Observatory (public domain): http://earthobservatory.nasa.gov/  Landsat: http://landsat.visibleearth.nasa.gov/  USGS EROS (Earth Resources Observatory and Science (EROS) Center) (public domain): http://eros.usgs.gov/#  Natural Earth (public domain): http://www.naturalearthdata.com | Thank you for your valuable feedback. After carefully reviewing your comment, we have decided to **remove Figure S1** from the manuscript, as it does not provide essential value to the overall message of the paper. The content can be understood without the inclusion of this figure, and we feel that removing it will address the copyright concerns raised. |
| 6 | If the reviewer comments include a recommendation to cite specific previously published works, please review and evaluate these publications to determine whether they are relevant and should be cited. There is no requirement to cite these works unless the editor has indicated otherwise. | Thank you so much, this is well noted |

References

1.	Ministry of Health Dodoma, M.o.H.Z., National Bureau of Statistics Dodoma,Office of Chief Government Statistician Zanzibar, *Tanzania Demographic Survey and Malaria Indicator Survey,*. 2023.

2.	Afnan-Holmes, H., et al., *Tanzania's countdown to 2015: an analysis of two decades of progress and gaps for reproductive, maternal, newborn, and child health, to inform priorities for post-2015.* Lancet Glob Health, 2015. **3**(7): p. e396-409.

3.	Bundala, F.A., et al., *Availability and functioning of neonatal care units and neonatal mortality across five regions in Tanzania: a baseline cross-sectional study in 127 CEmONC health facilities.* BMJ Glob Health, 2025. **10**(5).

4.	Kamuyu, R., et al., *Investment case for small and sick newborn care in Tanzania: systematic analyses.* BMC Pediatr, 2023. **23**(Suppl 2): p. 632.

5.	Kiwanuka, A., H.C. Kimaro, and W. Senyoni, *Analysis of the Acceptance Process of District Health Information Systems (DHIS) for Vertical Health Programmes: A Case Study of TB, HIV/AIDS and Malaria Programmes in Tanzania.* The Electronic Journal of Information Systems in Developing Countries, 2017. **70**(1): p. 1-14.

6.	Rumisha, S.F., et al., *Data quality of the routine health management information system at the primary healthcare facility and district levels in Tanzania.* BMC Med Inform Decis Mak, 2020. **20**(1): p. 340.

7.	Braa, J., A. Heywood, and S. Sahay, *Improving quality and use of data through data-use workshops: Zanzibar, United Republic of Tanzania.* Bull World Health Organ, 2012. **90**(5): p. 379-84.

8.	Shabani, J., et al., *Quality of reporting and trends of emergency obstetric and neonatal care indicators: an analysis from Tanzania district health information system data between 2016 and 2020.* BMC Pregnancy Childbirth, 2023. **23**(1): p. 716.

9.	Shabani, J., et al., *Neonatal indicator data in Tanzania District Health Information System: evaluation of availability and quality of selected newborn indicators, 2015-2022.* BMC Pediatr, 2025. **23**(Suppl 2): p. 658.

10.	Unkels, R., et al., *Caught in the data quality trap: A case study from the evaluation of a new digital technology supporting routine health data collection in Southern Tanzania*. 2023.

11.	UNICEF, *WHO technical consultation on Newborn Indicators.* 2014.

12.	Adane, A., et al., *Routine health management information system data in Ethiopia: consistency, trends, and challenges.* Glob Health Action, 2021. **14**(1): p. 1868961.

13.	WHO, *WHO data quality review: Module 2 Desk review of data quality*. 2017.

14.	Day, L.T., et al., *Labour and delivery ward register data availability, quality, and utility - Every Newborn - birth indicators research tracking in hospitals (EN-BIRTH) study baseline analysis in three countries.* BMC Health Serv Res, 2020. **20**(1): p. 737.

15.	Githinji, S., et al., *Completeness of malaria indicator data reporting via the District Health Information Software 2 in Kenya, 2011-2015.* Malar J, 2017. **16**(1): p. 344.

16.	Bhattacharya, A.A., et al., *Quality of routine facility data for monitoring priority maternal and newborn indicators in DHIS2: A case study from Gombe State, Nigeria.* PLoS One, 2019. **14**(1): p. e0211265.

17.	J.H.Lungo, *The reliability and usability of district health information software: case studies from Tanzania.* Tanzania Journal of Health Research, 2008.

18.	Nsaghurwe, A., et al., *One country's journey to interoperability: Tanzania's experience developing and implementing a national health information exchange.* BMC Med Inform Decis Mak, 2021. **21**(1): p. 139.

19.	Sukums, F., et al., *Avoiding pitfalls: Key insights and lessons learnt from customizing and rolling out a national web‐based system in Tanzania.* The Electronic Journal of Information Systems in Developing Countries, 2021. **87**(6).
