# Supplementary material for: Counting missing babies in Tanzania: Neonatal mortality data quality from Tanzania’s District Health Information System across 28 Regional and 7 Tertiary hospitals (2015–2024)
Source: PLoS One. 2026 Jul 23;21(7):e0348874. doi: 10.1371/journal.pone.0348874 (PMC13395327; doi:10.1371/journal.pone.0348874)
Supplement: Peer review history

# Peer review: Decision Letter 0

**DOI:** 10.1371/journal.pone.0348874.r001

**Authors:** Sabita Tuladhar

13 Jan 2026

PONE-D-25-58082

Counting missing babies in Tanzania: Neonatal mortality data quality from Tanzania's District Health Information System across 28 Regional and 7 Tertiary hospitals (2015-2024)

PLOS One

Dear Dr. Shabani,

Thank you for submitting your manuscript to PLOS ONE. After careful consideration, we feel that it has merit but does not fully meet PLOS ONE’s publication criteria as it currently stands. Therefore, we invite you to submit a revised version of the manuscript that addresses the points raised during the review process.

Please submit your revised manuscript by Feb 27 2026 11:59PM. If you will need more time than this to complete your revisions, please reply to this message or contact the journal office at plosone@plos.org. When you're ready to submit your revision, log on to https://www.editorialmanager.com/pone/ and select the 'Submissions Needing Revision' folder to locate your manuscript file.

Please include the following items when submitting your revised manuscript:

- A letter that responds to each point raised by the academic editor and reviewer(s). You should upload this letter as a separate file labeled 'Response to Reviewers'.
- A marked-up copy of your manuscript that highlights changes made to the original version. You should upload this as a separate file labeled 'Revised Manuscript with Track Changes'.
- An unmarked version of your revised paper without tracked changes. You should upload this as a separate file labeled 'Manuscript'.

If you would like to make changes to your financial disclosure, please include your updated statement in your cover letter. Guidelines for resubmitting your figure files are available below the reviewer comments at the end of this letter.

If applicable, we recommend that you deposit your laboratory protocols in protocols.io to enhance the reproducibility of your results. Protocols.io assigns your protocol its own identifier (DOI) so that it can be cited independently in the future. For instructions see: https://journals.plos.org/plosone/s/submission-guidelines#loc-laboratory-protocols. Additionally, PLOS ONE offers an option for publishing peer-reviewed Lab Protocol articles, which describe protocols hosted on protocols.io. Read more information on sharing protocols at https://plos.org/protocols?utm_medium=editorial-email&utm_source=authorletters&utm_campaign=protocols.

We look forward to receiving your revised manuscript.

Kind regards,

Sabita Tuladhar, PhD, MHealSc, MA

Academic Editor

PLOS One

Journal Requirements:

When submitting your revision, we need you to address these additional requirements.

1. Please ensure that your manuscript meets PLOS ONE's style requirements, including those for file naming. The PLOS ONE style templates can be found at

https://journals.plos.org/plosone/s/file?id=wjVg/PLOSOne_formatting_sample_main_body.pdf and

https://journals.plos.org/plosone/s/file?id=ba62/PLOSOne_formatting_sample_title_authors_affiliations.pdf.

2. We note that the grant information you provided in the ‘Funding Information’ and ‘Financial Disclosure’ sections do not match.

When you resubmit, please ensure that you provide the correct grant numbers for the awards you received for your study in the ‘Funding Information’ section.

3. We noted in your submission details that a portion of your manuscript may have been presented or published elsewhere. [We have one figure- S2 Fig. Health system and process of data collation in District Health Information System 2 Tanzania. This figure has been adapted from Shabani et.al BMC Paediatrics (2023)

https://bmcpediatr.biomedcentral.com/articles/10.1186/s12887-025-05417-x] Please clarify whether this [conference proceeding or publication] was peer-reviewed and formally published. If this work was previously peer-reviewed and published, in the cover letter please provide the reason that this work does not constitute dual publication and should be included in the current manuscript.

4. Your ethics statement should only appear in the Methods section of your manuscript. If your ethics statement is written in any section besides the Methods, please move it to the Methods section and delete it from any other section. Please ensure that your ethics statement is included in your manuscript, as the ethics statement entered into the online submission form will not be published alongside your manuscript.

5. We note that Figure S1 in your submission contain [map/satellite] images which may be copyrighted. All PLOS content is published under the Creative Commons Attribution License (CC BY 4.0), which means that the manuscript, images, and Supporting Information files will be freely available online, and any third party is permitted to access, download, copy, distribute, and use these materials in any way, even commercially, with proper attribution. For these reasons, we cannot publish previously copyrighted maps or satellite images created using proprietary data, such as Google software (Google Maps, Street View, and Earth). For more information, see our copyright guidelines: http://journals.plos.org/plosone/s/licenses-and-copyright.

We require you to either (1) present written permission from the copyright holder to publish these figures specifically under the CC BY 4.0 license, or (2) remove the figures from your submission:

a. You may seek permission from the original copyright holder of Figure S1 to publish the content specifically under the CC BY 4.0 license.

We recommend that you contact the original copyright holder with the Content Permission Form (http://journals.plos.org/plosone/s/file?id=7c09/content-permission-form.pdf) and the following text:

“I request permission for the open-access journal PLOS ONE to publish XXX under the Creative Commons Attribution License (CCAL) CC BY 4.0 (http://creativecommons.org/licenses/by/4.0/). Please be aware that this license allows unrestricted use and distribution, even commercially, by third parties. Please reply and provide explicit written permission to publish XXX under a CC BY license and complete the attached form.”

Please upload the completed Content Permission Form or other proof of granted permissions as an "Other" file with your submission.

In the figure caption of the copyrighted figure, please include the following text: “Reprinted from [ref] under a CC BY license, with permission from [name of publisher], original copyright [original copyright year].”

b. If you are unable to obtain permission from the original copyright holder to publish these figures under the CC BY 4.0 license or if the copyright holder’s requirements are incompatible with the CC BY 4.0 license, please either i) remove the figure or ii) supply a replacement figure that complies with the CC BY 4.0 license. Please check copyright information on all replacement figures and update the figure caption with source information. If applicable, please specify in the figure caption text when a figure is similar but not identical to the original image and is therefore for illustrative purposes only.

The following resources for replacing copyrighted map figures may be helpful:

USGS National Map Viewer (public domain): http://viewer.nationalmap.gov/viewer/

The Gateway to Astronaut Photography of Earth (public domain): http://eol.jsc.nasa.gov/sseop/clickmap/

Maps at the CIA (public domain): https://www.cia.gov/library/publications/the-world-factbook/index.html and https://www.cia.gov/library/publications/cia-maps-publications/index.html

NASA Earth Observatory (public domain): http://earthobservatory.nasa.gov/

Landsat: http://landsat.visibleearth.nasa.gov/

USGS EROS (Earth Resources Observatory and Science (EROS) Center) (public domain): http://eros.usgs.gov/#

Natural Earth (public domain): http://www.naturalearthdata.com/

6. If the reviewer comments include a recommendation to cite specific previously published works, please review and evaluate these publications to determine whether they are relevant and should be cited. There is no requirement to cite these works unless the editor has indicated otherwise.

[Note: HTML markup is below. Please do not edit.]

Reviewers' comments:

Reviewer's Responses to Questions

**Comments to the Author**

1. Is the manuscript technically sound, and do the data support the conclusions?

The manuscript must describe a technically sound piece of scientific research with data that supports the conclusions. Experiments must have been conducted rigorously, with appropriate controls, replication, and sample sizes. The conclusions must be drawn appropriately based on the data presented.

Reviewer #1: Yes

Reviewer #2: Yes

**********

2. Has the statistical analysis been performed appropriately and rigorously?

Reviewer #1: I Don't Know

Reviewer #2: No

**********

3. Have the authors made all data underlying the findings in their manuscript fully available?

The PLOS Data policy requires authors to make all data underlying the findings described in their manuscript fully available without restriction, with rare exception (please refer to the Data Availability Statement in the manuscript PDF file). The data should be provided as part of the manuscript or its supporting information, or deposited to a public repository. For example, in addition to summary statistics, the data points behind means, medians and variance measures should be available. If there are restrictions on publicly sharing data—e.g. participant privacy or use of data from a third party—those must be specified.

Reviewer #1: No

Reviewer #2: Yes

**********

4. Is the manuscript presented in an intelligible fashion and written in standard English?

PLOS ONE does not copyedit accepted manuscripts, so the language in submitted articles must be clear, correct, and unambiguous. Any typographical or grammatical errors should be corrected at revision, so please note any specific errors here.

Reviewer #1: Yes

Reviewer #2: No

**********

5. Review Comments to the Author

Please use the space provided to explain your answers to the questions above. You may also include additional comments for the author, including concerns about dual publication, research ethics, or publication ethics. (Please upload your review as an attachment if it exceeds 20,000 characters)

Reviewer #1: Thank you for the opportunity to review this manuscript. This paper is unique and important, particularly in the current landscape of declining donor funding for large-scale surveys and the limited attention given to the use of mortality data from routine management information systems.

Comments:

1. Page 3: The manuscript reports NMR estimates from the 2010 TDHS and “2025/16 TDHS.” Please confirm whether the year 2025/16 is correct.

2. Page 12: The authors state that “the analysis showed improvement in the number of facilities reporting mortality data from 2015 to 2024.” The actual number of reporting facilities increased, but how do the authors conclude that reporting improved? Please clarify the basis for this interpretation.

3. Use of abbreviations: Abbreviation use can be strengthened throughout the paper. For example, NEST360 should be expanded when first mentioned, as well as CPAP (Page 3, Line 76).

4. Methods section:

o How did the authors access the data used in the analysis?

o Is this data accessible to other users/researchers?

o In what format or template was the dataset made available or downloaded for analysis?

5. For international readers, please include a brief description of the Neonatal Inpatient Data (NID) from NEST360. What type of data or dataset does it include?

6. Results section:

In subsection “(1) Availability of neonatal mortality data” and Table 1, the information presented does not reflect the study findings, as the analysis focuses on 35 hospitals. This content seems more appropriate for the Background section. The Results section should present findings only from the 35 hospitals to avoid confusion and ensure alignment with the study objectives.

7. Page 12, line 272. Does an increase in the number of reported neonatal deaths necessarily indicate improvement? Please clarify the interpretation and its implications.

8. The manuscript is heavily data-focused without adequate programmatic context, which may limit its readability for program managers or practitioners. Consider adding:

o An overview of neonatal programs in Tanzania;

o Data quality improvement activities implemented (e.g., service provider or medical recorder training, routine data quality assessments);

o Any existing MPDSR or neonatal mortality surveillance systems.

9. Discussion:

The discussion can be strengthened by adding contextual information, such as comparisons with other countries—preferably within Africa or other LMICs. If no comparable studies exist, that should be stated as an important discussion point.

Additionally, how do neonatal mortality data compare with maternal or other mortality indicators reported in DHIS2? What is known about the overall quality of DHIS2 data in Tanzania?

Strengthening routine data systems is crucial, especially given current reductions in donor-funded programs such as DHS. Based on the analysis, what specific recommendations can the authors offer for improving data quality?

10. Limitations:

The limitations section should be revisited. The analysis could potentially have been disaggregated by public versus private hospitals. Are there additional limitations related to the use of historical data or known data quality issues?

Reviewer #2: Dear editor,

Thank you for involving me in the peer review of this manuscript. I have provided the authors with the detailed review report below and invite authors to address all the points raised during my review. Regards.

Overall assessment

This is a timely, policy-relevant, and important manuscript addressing a critical gap in neonatal mortality surveillance in Tanzania. The focus on secondary and tertiary hospitals, the long time span (2015–2024), and the triangulation across DHIS2, DHS, UN-IGME, and NEST360 NID are major strengths. The paper convincingly demonstrates substantial under-capture and inconsistency in DHIS2 neonatal mortality reporting.

However, the manuscript would benefit from clearer conceptual framing, stronger justification of methodological choices, more cautious interpretation of comparisons, and clearer articulation of implications for HMIS strengthening. Several areas also require clarification to avoid misinterpretation by readers unfamiliar with DHIS2 or inpatient vs population-based mortality metrics.

Major comments

1. Clarify the conceptual distinction between inpatient neonatal mortality and population-based neonatal mortality

Issue:

Throughout the manuscript, inpatient NMRs from DHIS2 are repeatedly compared to DHS and UN-IGME population-based NMRs. While the authors acknowledge this difference, the conceptual implications are not sufficiently foregrounded.

Why this matters:

Readers may incorrectly interpret discrepancies as purely data quality problems, rather than also reflecting fundamentally different denominators, case-mix, and risk profiles.

Suggestions:

Explicitly state early (Introduction or Methods) that:

DHIS2 hospital NMRs are not expected to match population-based NMRs.

Referral hospitals should, if anything, have higher inpatient mortality, not lower.

Consider adding a conceptual figure or box explaining:

Expected direction of bias when comparing DHIS2 vs DHS/UN estimates.

Strengthen the argument that the direction and magnitude of discrepancy strongly indicate underreporting, even after accounting for these differences.

2. Justify the choice of ±33% threshold for internal consistency more explicitly

Issue:

The ±33% WHO threshold is applied, but the rationale and appropriateness for neonatal mortality in referral hospitals is not fully discussed.

Why this matters:

Given the high year-to-year variability expected in hospital case-mix, readers may question whether ±33% is too strict or too lenient.

Suggestions:

Provide a short justification explaining why this threshold is appropriate for:

High-volume referral hospitals

Mortality indicators rather than service-delivery indicators

Consider a sensitivity analysis using a wider threshold (e.g. ±50%) or explicitly acknowledge this as a limitation.

3. Interpretation of very high inpatient NMRs needs strengthening

Issue:

Some hospitals report inpatient NMRs exceeding 100 per 1,000 live births, which is briefly noted but not deeply explored.

Why this matters:

Such values could reflect:

Severe referral bias

Denominator problems (e.g. births counted outside neonatal units)

Data entry or classification errors

Suggestions:

Expand the Discussion to explicitly address:

Whether these extreme values are plausible

Whether they suggest numerator inflation, denominator undercounting, or both

Clarify whether live births used as denominators include:

Only births occurring in the hospital

Or also referrals admitted post-birth

4. Completeness analysis would benefit from clearer interpretation

Issue:

The manuscript reports completeness rigorously but stops short of interpreting what level of completeness is operationally acceptable for mortality surveillance.

Suggestions:

Explicitly state why >80% completeness was chosen as a benchmark.

Discuss implications of <60% completeness for:

Trend analysis

Facility benchmarking

National aggregation

Consider classifying facilities into performance tiers (e.g. high, moderate, low completeness) and summarizing their characteristics.

5. Strengthen the policy and systems implications

Issue:

The Conclusions and Discussion point to “improving reporting” but remain relatively general.

Suggestions:

Be more explicit about actionable system-level recommendations, such as:

Linking DHIS2 neonatal death reporting to routine mortality audit processes

Harmonising DHIS2 indicators with NEST360/NID definitions

Introducing automated plausibility checks within DHIS2

Clarify how high-performing RRHs could be used as:

Learning sites

Mentorship hubs

Sentinel surveillance facilities

Minor comments and technical suggestions

Terminology

Use “live births” consistently (avoid alternating with “livebirths”).

Clarify when “neonatal mortality rate” refers specifically to inpatient mortality.

Grammar and clarity

Several sentences are long and could be tightened, particularly in the Introduction and Discussion.

Example:

“Although the percentages of births in health facilities has increased…”

→ should be “have increased”.

Figures and tables

Ensure all figures clearly label:

Denominators

Facility types

Data sources

Table 3 is very dense; consider splitting into:

Deaths & births

NMR & consistency metrics

Methods transparency

Specify whether zero values in DHIS2 were treated as:

True zero deaths

Or missing reports

Clarify how missing live birth data were handled in NMR calculations.

Ethics statement

Well written and appropriate; no major concerns.

Specific questions for the authors (points needing clarification)

Denominator definition:

Are live births in DHIS2 limited to births occurring within the facility, or do they include births occurring elsewhere but later admitted?

Zero reporting:

How did you distinguish between true zero neonatal deaths and months with missing reports recorded as zero?

Referral bias:

Did you explore whether hospitals with higher completeness also had higher reported NMRs, suggesting more realistic reporting?

NEST360 comparison:

Were case definitions for neonatal death fully harmonised between DHIS2 and NID, particularly regarding timing (early vs late neonatal death)?

Private sector role:

Given the “Others” category contributes substantially to deaths, what are the implications for national neonatal mortality surveillance beyond public hospitals?

Overall recommendation

This manuscript addresses a critical gap in neonatal mortality measurement and has strong potential to influence health information system strengthening in Tanzania and similar settings. With clearer framing, strengthened interpretation, and more explicit policy implications, it would make a valuable contribution to the literature.

**********

6. PLOS authors have the option to publish the peer review history of their article (what does this mean?). If published, this will include your full peer review and any attached files.

If you choose “no”, your identity will remain anonymous but your review may still be made public.

**Do you want your identity to be public for this peer review?**  For information about this choice, including consent withdrawal, please see our Privacy Policy.

Reviewer #1: **Yes:** Sabita Tuladhar

Reviewer #2: No

**********

[NOTE: If reviewer comments were submitted as an attachment file, they will be attached to this email and accessible via the submission site. Please log into your account, locate the manuscript record, and check for the action link "View Attachments". If this link does not appear, there are no attachment files.]

To ensure your figures meet our technical requirements, please review our figure guidelines: https://journals.plos.org/plosone/s/figures

You may also use PLOS’s free figure tool, NAAS, to help you prepare publication quality figures: https://journals.plos.org/plosone/s/figures#loc-tools-for-figure-preparation.

NAAS will assess whether your figures meet our technical requirements by comparing each figure against our figure specifications.

# Author comment: Author response to Decision Letter 1

**DOI:** 10.1371/journal.pone.0348874.r002

9 Apr 2026

Thank you for the opportunity to submit a revised version of our manuscript. We are deeply appreciative of the reviewers for their thoughtful comments and suggestions. These specific comments enabled us to improve and strengthen the manuscript. Please find below our response to the reviewer’s comments.

Thank you for your consideration of this re-submission and please let us know if more information is required.

Attachment Submitted filename: Response to reviewers.docx

# Peer review: Decision Letter 1

**DOI:** 10.1371/journal.pone.0348874.r003

**Authors:** Sabita Tuladhar, Sabita Tuladhar

23 Apr 2026

Counting missing babies in Tanzania: Neonatal mortality data quality from Tanzania's District Health Information System across 28 Regional and 7 Tertiary hospitals (2015-2024)

PONE-D-25-58082R1

Dear Dr. Shabani,

We’re pleased to inform you that your manuscript has been judged scientifically suitable for publication and will be formally accepted for publication once it meets all outstanding technical requirements.

Within one week, you’ll receive an e-mail detailing the required amendments. When these have been addressed, you’ll receive a formal acceptance letter and your manuscript will be scheduled for publication.

An invoice will be generated when your article is formally accepted. Please note, if your institution has a publishing partnership with PLOS and your article meets the relevant criteria, all or part of your publication costs will be covered. Please make sure your user information is up-to-date by logging into Editorial Manager at Editorial Manager® and clicking the ‘Update My Information' link at the top of the page. For questions related to billing, please contact billing support.

If your institution or institutions have a press office, please notify them about your upcoming paper to help maximize its impact. If they’ll be preparing press materials, please inform our press team as soon as possible -- no later than 48 hours after receiving the formal acceptance. Your manuscript will remain under strict press embargo until 2 pm Eastern Time on the date of publication. For more information, please contact onepress@plos.org.

Kind regards,

Sabita Tuladhar, PhD, MHealSc, MA

Academic Editor

PLOS One

Additional Editor Comments (optional):

Thank you for carefully addressing the reviewers’ comments. Figures 1, 2, 3, and 4 are blurred and lack adequate visibility. These figures should be corrected during the proofing stage or through other means, as per the journal’s decision. In addition, the authors should review the use of abbreviations and punctuation throughout the manuscript.

Reviewers' comments:

Reviewer's Responses to Questions

**Comments to the Author**

1. If the authors have adequately addressed your comments raised in a previous round of review and you feel that this manuscript is now acceptable for publication, you may indicate that here to bypass the “Comments to the Author” section, enter your conflict of interest statement in the “Confidential to Editor” section, and submit your "Accept" recommendation.

Reviewer #1: All comments have been addressed

Reviewer #2: All comments have been addressed

**********

2. Is the manuscript technically sound, and do the data support the conclusions?

The manuscript must describe a technically sound piece of scientific research with data that supports the conclusions. Experiments must have been conducted rigorously, with appropriate controls, replication, and sample sizes. The conclusions must be drawn appropriately based on the data presented.

Reviewer #1: Yes

Reviewer #2: Yes

**********

3. Has the statistical analysis been performed appropriately and rigorously?

Reviewer #1: Yes

Reviewer #2: Yes

**********

4. Have the authors made all data underlying the findings in their manuscript fully available?

The PLOS Data policy requires authors to make all data underlying the findings described in their manuscript fully available without restriction, with rare exception (please refer to the Data Availability Statement in the manuscript PDF file). The data should be provided as part of the manuscript or its supporting information, or deposited to a public repository. For example, in addition to summary statistics, the data points behind means, medians and variance measures should be available. If there are restrictions on publicly sharing data—e.g. participant privacy or use of data from a third party—those must be specified.

Reviewer #1: Yes

Reviewer #2: Yes

**********

5. Is the manuscript presented in an intelligible fashion and written in standard English?

PLOS ONE does not copyedit accepted manuscripts, so the language in submitted articles must be clear, correct, and unambiguous. Any typographical or grammatical errors should be corrected at revision, so please note any specific errors here.

Reviewer #1: Yes

Reviewer #2: Yes

**********

6. Review Comments to the Author

Please use the space provided to explain your answers to the questions above. You may also include additional comments for the author, including concerns about dual publication, research ethics, or publication ethics. (Please upload your review as an attachment if it exceeds 20,000 characters)

Reviewer #1: This is a unique paper and highly relevant for LMICs that are increasingly investing in non‑routine systems to monitor mortality data. The recommendations will be extremely useful for improving the quality of mortality reporting through routine systems.

The authors are encouraged to review abbreviations and punctuation throughout the manuscript for consistency and clarity.

Reviewer #2: Dear authors, thank you for addressing my comments. I endorse acceptance of this work for publication from my side. However, the figures 1, 2, 3 and 4 are blurred and lack visibility. I recommend them to be corrected during proofing or other means as per the journal decision.

**********

7. PLOS authors have the option to publish the peer review history of their article (what does this mean?). If published, this will include your full peer review and any attached files.

If you choose “no”, your identity will remain anonymous but your review may still be made public.

**Do you want your identity to be public for this peer review?**  For information about this choice, including consent withdrawal, please see our Privacy Policy.

Reviewer #1: **Yes:** Sabita Tuladhar

Reviewer #2: No

**********

# Editor report: Acceptance letter

**DOI:** 10.1371/journal.pone.0348874.r004

**Authors:** Sabita Tuladhar, Sabita Tuladhar

PONE-D-25-58082R1

PLOS One

Dear Dr. Shabani,

I'm pleased to inform you that your manuscript has been deemed suitable for publication in PLOS One. Congratulations! Your manuscript is now being handed over to our production team.

At this stage, our production department will prepare your paper for publication. This includes ensuring the following:

* All references, tables, and figures are properly cited

* All relevant supporting information is included in the manuscript submission,

* There are no issues that prevent the paper from being properly typeset

You will receive further instructions from the production team, including instructions on how to review your proof when it is ready. Please keep in mind that we are working through a large volume of accepted articles, so please give us a few days to review your paper and let you know the next and final steps.

Lastly, if your institution or institutions have a press office, please let them know about your upcoming paper now to help maximize its impact. If they'll be preparing press materials, please inform our press team within the next 48 hours. Your manuscript will remain under strict press embargo until 2 pm Eastern Time on the date of publication. For more information, please contact onepress@plos.org.

You will receive an invoice from PLOS for your publication fee after your manuscript has reached the completed accept phase. If you receive an email requesting payment before acceptance or for any other service, this may be a phishing scheme. Learn how to identify phishing emails and protect your accounts at https://explore.plos.org/phishing.

If we can help with anything else, please email us at customercare@plos.org.

Thank you for submitting your work to PLOS ONE and supporting open access.

Kind regards,

PLOS ONE Editorial Office Staff

on behalf of

Dr. Sabita Tuladhar

Academic Editor

PLOS One
